# Setting a standard for low reading proficiency: A comparison of the bookmark procedure and constrained mixture Rasch model

**DOI:** 10.1371/journal.pone.0257871

**Published:** 2021-11-29

**Authors:** Tabea Feseker, Timo Gnambs, Cordula Artelt

**Affiliations:** LIfBi–Leibniz Institute for Educational Trajectories, Bamberg, Germany; University of Copenhagen, DENMARK

## Abstract

In order to draw pertinent conclusions about persons with low reading skills, it is essential to use validated standard-setting procedures by which they can be assigned to their appropriate level of proficiency. Since there is no standard-setting procedure without weaknesses, external validity studies are essential. Traditionally, studies have assessed validity by comparing different judgement-based standard-setting procedures. Only a few studies have used model-based approaches for validating judgement-based procedures. The present study addressed this shortcoming and compared agreement of the cut score placement between a judgement-based approach (i.e., Bookmark procedure) and a model-based one (i.e., constrained mixture Rasch model). This was performed by differentiating between individuals with low reading proficiency and those with a functional level of reading proficiency in three independent samples of the German National Educational Panel Study that included students from the ninth grade (*N* = 13,897) as well as adults (*N*s = 5,335 and 3,145). The analyses showed quite similar mean cut scores for the two standard-setting procedures in two of the samples, whereas the third sample showed more pronounced differences. Importantly, these findings demonstrate that model-based approaches provide a valid and resource-efficient alternative for external validation, although they can be sensitive to the ability distribution within a sample.

## Introduction

In order to justify high-stakes decisions and draw relevant conclusions about persons with low reading skills, stringent criteria are required to validly classify individuals as being at risk. Therefore, it is essential to use validated standard-setting procedures that allow for distinguishing between groups of individuals with different reading proficiency levels and facilitate a criterion-referenced interpretation of a person’s skills at a given proficiency level. In this context, validation studies play a crucial role in validating the chosen cut scores [1, 2]. In practice, the Bookmark procedure [3] has become one of the most popular standard-setting procedures because the established proficiency levels offer criterion validity through an iterative process of judging test content in combination with descriptors of different proficiency levels that were developed prior to their empirical implementation [4–6]. Many experts in recent years have emphasized that this judgmental task is also a central concern for the validity of the Bookmark procedure, since the choice of the cut scores can be influenced by a number of factors, such as the composition and training of the panelists, the formulation of the proficiency level descriptors, the choice of the response probability, or the range and number of the test items, to name a few [4, 5, 7]. A number of studies addressed this concern by examining whether the cut scores derived by the Bookmark procedure correspond to those derived by other standard-setting procedures. However, most of these studies compared the Bookmark procedure with standard-setting procedures of the same kind, that is, procedures requiring subjective judgment by content experts (e.g., Angoff procedure) to establish different proficiency levels [8–15]. A major weakness of these studies is that each of the examined procedures is influenced by the subjectivity of the panelists and is highly resource-intensive (i.e., costly in terms of time and personnel). In the search for alternatives, validation studies have considered other standard-setting procedures, which allow a model-based proficiency scaling based on empirical information from respondents’ item response patterns [16–21], such as latent class analysis or mixture Rasch modeling. However, despite its increasing application for standard setting, to the best of our knowledge, Jiao and colleagues [17] presented the only study that examined the consistency in the assignment of individuals to a certain proficiency level, between the Bookmark procedure and a model-based approach using simulated data.

The present study addresses this shortcoming and presents a validity study that investigates validity arguments for the interpretation of cut scores applied for the purpose of standard setting in a German large-scale assessment, the National Educational Panel Study (NEPS; [22]), to differentiate between low-literate and functionally literate adolescents and adults. Unlike other large-scale assessments, the provision of proficiency levels was initially not intended for the NEPS assessments [23]. However, the Bookmark procedure was applied afterwards in order to be able to define low literacy and to investigate the causes for and changes among adolescents and adults with low literacy [24]. The purpose of this study is, therefore, to compare the derived cut scores from the Bookmark procedure with a constrained mixture Rasch model approach in three independent samples of the NEPS. More generally, we aim at advancing our knowledge about the conclusions that can be drawn from the standard-setting procedures and of the strengths and limitations of both procedures.

We begin with a brief overview of the two standard-setting approaches, followed by the reasons for cross-validating the two approaches, before presenting the results of our empirical study.

### Judgement-based and model-based standard-setting approaches

In practice, two classes of standard-setting approaches are used for setting proficiency levels. The first class incorporates subjective expert judgments either on test items (item-centered approaches), such as the Bookmark procedure [3, 6] and Angoff procedure [25], or test takers (examinee-centered approaches), such as the Borderline Group [26]. Among those procedures, the Bookmark procedure [3, 6] is one of the most popular methods for setting standards [4, 5]. It involves several steps: Panelists are asked to make judgements about whether a test taker with a given proficiency is likely to correctly solve different test items. To make these judgements, the required material must be composed in the first step: this includes an ordered item booklet (OIB), the development of proficiency level descriptors, and the selection of the response probability value (RP value). The OIB includes the test items, which are arranged from the easiest to the hardest using the item difficulty parameters gained from the item response theory (IRT) model. The response probability “is used to determine the scale locations for the items and hence their ordering within the OIB” [4 p7], and is typically set at a probability of 67% [27, 28]. The proficiency level descriptors include a verbal description of the skills that test takers are expected to have at a given proficiency level and are often formulated as “can-do” statements. These are used to guide the discussion of the test content, as well as for communicating differences between the proficiency levels later on [29]. In the second step, content experts, or the so-called panelists, are selected and trained. Finally, the panelists place cut scores (i.e., “bookmarks”) between those items which, in their view, define the boundary between two proficiency levels. This step works in an iterative manner; the panelists discuss the items and set their bookmarks in subsequent rounds, and the process usually involves three rounds. After each round they receive feedback on their decisions and are encouraged to discuss this feedback with the other panelists. At the beginning of the third round, panelists are additionally provided with information about the percentage of individuals falling into a certain proficiency level (‘impact data’). After the panelists set the final bookmarks, the recommended cut scores for each proficiency level are calculated, often based on the mean or median of the judgements [3, 6, 26].

The second class of standard-setting procedures includes model-based classifications in order to assign individuals to a certain proficiency level [16–21]. Two popular methods are latent class analysis (LCA) [30] and mixture Rasch modeling (MRM; [31–33]), which use information from respondents’ item response patterns to identify distinct proficiency classes. With LCA, individuals are grouped into a set of latent classes (i.e., proficiency levels) in such a way that response patterns are maximally homogeneous for all persons within the same class and maximally heterogeneous between classes. The dichotomous form of the LCA is expressed as

$$P(x_{vi}=1)=\sum_{g=1}^{G}\pi_{g}\;\pi_{ig}$$ (1)

where *P*(*x*_*vi*_ = 1) is the response probability for an individual *v* to solve item *i*, *π*_*g*_ is the relative class size, *and π*_*ig*_ is the response probability for item *i* in class *g* (class-specific response probability). In contrast, MRM combines LCA and the Rasch analysis [31–33] by assuming that the Rasch model holds within each class but item difficulty parameters can vary between classes. Thus, MRM allows for estimating an individual’s latent ability and class membership. The dichotomous form of the MRM is expressed as

$$P(x_{vi}=1)=\sum_{g=1}^{G}\pi_{g}\frac{exp(\theta_{vg}-\sigma_{ig})}{1+exp(\theta_{vg}-\sigma_{ig})}$$ (2)

where *P*(*x*_*vi*_ = 1) is the response probability for an individual *v* to solve item *i*, *π*_*g*_ is the relative class size, *θ*_*vg*_ is the ability of an individual *v* given that he or she belongs to class *g*, and *σ*_*ig*_ is the difficulty parameter of item *i* in class *g* [31–33]. To establish proficiency levels with the LCA or MRM, the optimal number of classes is determined based on different fit indices [34–36], the quality of the classification with regard to classification uncertainty (e.g., entropy), and practical considerations (e.g., minimum class size). In addition, robustness checks that replicate the analysis in different subgroups (e.g., with a split-half validation procedure) can help in evaluating the robustness of the selected class solution. Finally, individuals are assigned to the proficiency level that matches their most probable class membership.

### The need for validation analyses

Given that empirical support for validity is central to the conclusions that can be drawn on the basis of the cut scores, a number of studies examined classification agreement between the cut scores resulting from different standard-setting procedures. With respect to the Bookmark procedure, existing studies have largely compared the results of the Bookmark procedure with other judgement-based standard-setting procedures. The results show that different procedures sometimes produce very different cut scores, and the Bookmark procedure tended to produce lower cut scores and, therefore, a lower percentage of individuals falling into the lowest proficiency level [9–15]. In contrast, relatively few studies have compared the results of judgment-based and model-based standard-setting procedures. For example, Brown [16] and Strickman [19] compared the level of agreement between the Angoff procedure and LCA and found agreement rates ranging from less than 60% to almost perfect agreement. Jiao and colleagues [17] used simulated data to compare the level of agreement obtained from the Bookmark procedure and MRM. They found an overall agreement of 86.3%, which ranged from 73.0% to 92.4% within the respective proficiency levels.

Thereby, cross-validation of a judgment-based standard-setting approach with a model-based approach, as carried out in the abovementioned studies, offers several advantages compared to cross-validation between two similar approaches. First, in judgment-based standard-setting procedures, the setting of cut scores is per se a subjective process, as the procedures incorporate subjective expert judgments on test items or test takers [8–15, 37]. Therefore, poor agreement between two judgement-based procedures may be attributable to errors associated with the subjectivity inherent in these procedures. Thus, a combination of judgement-based and model-based approaches offers greater independence of both approaches, as a model-based approach offers a higher degree of objectivity due to its reliance on item response patterns [16–21]. Second, judgement-based procedures are resource intensive; for example, in terms of cognitive complexity for panelists or the time required [1]. Thus, using model-based procedures instead of other judgement-based procedures for validation could provide a resource-efficient alternative. Third, presuming that there is satisfactory classification agreement between a judgement-based and a model-based procedure, model-based procedures offer straightforward ways to take account of the degree of classification uncertainty in further analyses. Considering the level of uncertainty is important; such as when reporting the aggregated percentages of individuals in different proficiency levels or when changes in proficiency levels are the focus of interest. Uncertainty in judgement-based procedures is commonly reported in the form of standard errors or sometimes confidence intervals of the derived cut scores [8, 9, 11–14, 37], but there are no clear recommendations on how to take uncertainties into account for further analyses. In contrast, one major strength of model-based standard-setting procedures is that individuals are not manifestly assigned to a particular proficiency level but, rather, belong to each proficiency level (i.e., each latent class) with a certain probability as suggested by the class-specific model probabilities [31–33]. Thus, the information on a person’s posterior class membership, and thus the level of uncertainty, can be included in further analyses so that classification error can be accounted for. Finally, not only do judgement-based procedures benefit from cross-validation with model-based procedures, but cross-validation is also worthwhile for the validation of model-based procedures. Model-based procedures provide limited insight into the proficiency differences between individuals at different levels. Although model-based procedures such as LCA or MRM can be used to draw conclusions about the proportion of individuals at different proficiency levels and about differences in the probability of solving the respective tasks between the different proficiency groups, they do not—unlike judgement-based procedures—allow a criteria-based interpretation of a person’s abilities at a particular proficiency level. Thus, not only can the communication of the proficiency levels be difficult, the interpretation may be misleading. If, for example, the proportion of test takers with low reading proficiency in the sample is very small and most of the respondents are highly skilled, then it is more likely that the lowest proficiency level will include persons who would actually be assigned to a higher proficiency level on the basis of their abilities [18]. Therefore, the combination of both approaches can provide more content-valid evidence support.

In summary, both approaches have specific strengths and limitations. Using a combination of judgement-based and model-based standard-setting procedures could increase validity support, as the strengths of one approach can compensate for the limitations of another approach. As empirical evidence for the applicability of both the approaches for validation purposes is rare, our study aims to compare the cut scores obtained from the Bookmark procedure and a variant of mixture Rasch modeling.

## Method

### Samples

This study involved the use of data from the German *National Educational Panel Study* (NEPS) that provides, among others, data on domain-specific competencies for different representative samples across the life course [22]. The present study focused on three samples of students and adults who participated in assessments of their reading competence. Sample 1 included a representative sample of students in lower secondary education “Starting Cohort Grade 9” (doi:10.5157/NEPS:SC4:10.0.0) who attended ninth grade across different schools in Germany (for details on the sampling procedure, see [38]). All the major school types except for special schools were considered: 22.15% went to lower secondary school track, 22.36% to intermediate secondary school track, 34.93% to academic school track, and 20.56% to other school branches (including comprehensive schools, Waldorf schools, and other kind of schools). The sample included 13,897 students (49.87% female) with a mean age of *M* = 15.61 years (*SD* = 0.63). About 12.62% had a non-German background, meaning that their first language was not German. All the participants were tested for reading competence in wave 2, 2011/12 [39], by trained interviewers in small groups at their respective schools.

Samples 2 and 3 comprised two representative (non-overlapping) samples of the NEPS “Starting Cohort Adults” (in the following referred to as “Adult Sample 1” and “Adult Sample 2,” doi:10.5157/NEPS:SC6:10.0.0), including adults living in private households in Germany who were born between 1944 and 1986 (for details on the sampling procedures, see [40]). The reading test was first administered in wave 3, 2010/11, to Adult Sample 1 [41]. The same test was administered to Adult Sample 2 in wave 5, 2012/13 [42]. The samples consisted of *N* = 5,335 (50.20% female) and *N* = 3,145 (49.00% female) respondents, respectively. The participants’ ages ranged from 24 to 69 years, with means of *M* = 47.66 years (*SD* = 10.91) and *M* = 49.37 years (*SD* = 11.40), respectively. The proportion of respondents with a non-German background was 5.70% and 8.11%, respectively. All the participants were individually tested by trained interviewers in their private homes. Further information on the data collection process is available on the project website (www.neps-data.de).

### Instruments

#### Testing reading competence

In order to obtain a coherent measurement of reading competence in different samples over the life course, age-appropriate tests were developed based on a common theoretical framework with a focus on the functional component of reading (literacy approach [23]). The NEPS reading framework distinguishes three main dimensions: text functions, cognitive requirements, and task formats. Each reading test consists of five texts with different functions that are of practical relevance for handling written texts in different and typical everyday situations: information texts, commenting texts or argumenting texts, literary texts, instruction text, and advertising text. The cognitive requirements refer to the process that participants must employ in order to solve the task and distinguish between (1) finding information in the text, (2) drawing text-related conclusions, and (3) reflecting and assessing (situation model). The majority of the items included multiple-choice response formats and were scored dichotomously. However, several items that were composed of a number of subtasks referring to a common stimulus (e.g., decision-making tasks, matching tasks) were aggregated to polytomous ‘super-items’ [43]. The different cognitive requirements and task types were applied in all five texts [23]. The reading test was identical for the two adult samples with a total of 30 items, but different from the reading test for the student sample with a total of 31 items. No common items were included in the two tests. The order of the items was identical for all the participants of a given sample. All the items were presented through a paper-and-pencil test with a maximum test duration of 28 minutes [39, 41, 42].

The responses of each test were scaled using a variant of a partial credit model [44]. For multiple choice items, a scoring of 0 point for an incorrect and 1 point for the correct response was applied, while for polytomous items a scoring of 0.5 points for each category was used [45]. For identification, the latent proficiency distribution was fixed to a mean of 0. Reading scores were estimated as weighted maximum likelihood estimates (WLE) [46]. The mean of the reading competence test in the student sample was *M* = -0.03 (*SD* = 1.26, *Min* = -4.75, *Max* = 3.30). The Adult Samples 1 and 2 had means of *M* = -0.04 (*SD* = 1.35, *Min* = -5.70, *Max* = 4.49) and *M* = -0.36 (SD = 1.38, *Min* = -5.24, *Max* = 4.43), respectively (also see S1 Fig).

#### Quality of the tests

In-depth psychometric evaluations of the reading tests are described in Haberkorn and colleagues [39], Hardt and colleagues [41], and Koller and colleagues [42]. The reported results showed that, despite their short lengths, the tests had good marginal reliabilities with .75, .72, and .74 in the student and two adult samples, respectively. The correlations between the item score and total score (i.e., item discrimination indices) exceeded .20 for all items, with mean correlations of .44, .42, and .45 in the three samples [39, 41, 42]. Item fit statistics in terms of standardized weighted mean square residuals (infit) fell within conventional thresholds of acceptable fit, that is, between 0.8 and 1.2 [47, 48]. Moreover, visual comparisons of the expected item characteristics curves (ICC) with the observed non-parametric ICCs showed satisfactory item fits in terms of curvature and monotony of the observed ICCs [49]. In addition, unidimensionality was examined by fitting three- and five-dimensional models representing the different cognitive requirements and text functions (see above) to the data. These analyses showed substantial correlations ranging from *r* = .75 to *r* = .98 between the latent dimensions indicating that a common latent proficiency described the data reasonably well. This was also corroborated by the adjusted Q3 statistic [50] that did not exceed .20 for any item pair (the largest absolute Q3s were .16, .17, and .17 in the student and the two adult samples) and, thus, supported the assumption of local item independency for the three reading tests. Furthermore, analyses of differential item functioning corroborated the test fairness of the administered reading tests for several subgroups such as gender, socioeconomic status, migration background, and school degree [39, 41, 42].

### Standard-setting procedures

#### Bookmark procedure

The Bookmark procedure was used to set proficiency levels allowing for differentiation between a low reading proficiency level and a functional level of reading proficiency, in order to investigate the causes for and changes among individuals with low reading proficiency [24]. The implementation of the Bookmark procedure included the following steps:

1. Development of Proficiency Level Descriptors: An expert group developed a process model that explicated difficulties low-literate readers might face during different stages of a reading process due to reader-related, text-related, and task-related factors. Based on the process model, difficulty-generating factors were established and translated into proficiency level descriptors (PLDs). The PLDs included specific “can-do” descriptions of what low-literate readers probably can do and probably cannot do, thus providing guidance for the Bookmark procedure (see [24] for further details).
2. OIB and RP value: The reading items of the respective test were arranged in an OIB according to their item difficulty derived from a unidimensional Rasch model [39, 41, 42], from the easiest to most difficult item. Given that partial credit items were aggregated to polytomous super-items in the scaling model, polytomous items each appeared in one location of the OIB with one corresponding item difficulty parameter. The RP value was set at 67%.
3. Panelist Composition: A group of eleven panelists were sampled from a pool of test developers and professionals working with large-scale assessments and reading comprehension tests in Germany.
4. Training of Panelists: In a workshop, the panelists were familiarized with the standard-setting concept (setting a cut score to differentiate low-literate and literate readers), the NEPS reading framework, the Bookmark procedure, and the PLDs. The panelists were invited to share their thoughts with the other panel members and to discuss emerging questions.
5. Implementation of the Bookmark procedure: The Bookmark procedure took place in three rounds [6]. In rounds 1 and 2, the panelists discussed each item presented in the OIB in small groups, using the PLDs. They evaluated the difficulty of each item and the proficiency required for a person to achieve a certain proficiency level, by repeatedly comparing the reading items with the PLDs. The basic question the panelists discussed was “Is it likely, i.e., is there a 67% chance, that a person with low reading proficiency will answer this item correctly?”. After the group completed their discussion, each panelist independently set a cut score (i.e., “bookmark”) in the OIB, defining the reading item that differentiates between low-literate and literate readers. Round 3 took place with the whole group of panelists. The panelists received impact data from round 2 on the percentage of individuals who fell into the low-literacy group at a given cut score. After discussing the impact data, the panelists were asked to reach consensus on the item that defines the boundary between the two proficiency levels. To calculate the cut score value needed to have a 67% chance of answering that reading item correctly, 0.701 logits were added to the item difficulty parameter of this item obtained from the scaling model. In the student sample, the items 1, 2, 5, 12, 13, and 15 were used within the Bookmark procedure to define low literacy, while items 1, 2, 3, 6, 7, 8, 9, 23 and 26 were used in the two adult samples. The final cut score values for distinguishing between low-literate and literate readers were -2.11 logits for the student sample and -1.74 logits for the two adult samples.

#### Constrained mixture Rasch model

Standard setting generally assumes unidimensionality for the entire population in order to define proficiency levels. This is the case, for example, with the Bookmark procedure, which assumes that the test fits a unidimensional Rasch model [3, 6]. As described above MRMs, in contrast, assume that persons belong to one of several latent classes in which the Rasch model holds within each class but item difficulty parameters can vary between classes [31–33]. In this case, the differences between individuals belonging to different latent classes are not only differences in proficiency levels, but also differences, for example, in strategies and skills to solve an item. Thus, not only would the individuals belonging to different latent classes not be comparable, but also the proficiency levels cannot be compared between the Bookmark procedure and MRM. To solve this problem, we constrained the item difficulty parameters to be equal across the classes. By imposing equality constraints, differences between classes can be explained by quantitative differences in the respondents’ abilities rather than qualitative differences in item functioning. Moreover, we assumed that the heterogeneity of respondents’ proficiencies could be accounted for by the set of latent classes alone without modeling additional variability within classes. Thus, in contrast to the modeling strategy from Rost and von Davier [31–33], we fixed the factor variances in each class to zero [51]. In this way, the latent trait distribution is represented by a set of discrete levels along the proficiency continuum with homogenous respondents within each class. Consequently, these classes reflect distinct reading proficiency levels similar to the assumptions of the Bookmark procedure. Therefore, to distinguish our modeling strategy from the MRM of Rost and von Davier [31–33], we refer to our model as the “constrained mixture Rasch model” (cMRM). In the supplementary material, we included item fit information contrasting the continuous trait Rasch models and categorical trait cMRMs. These analyses showed that both models exhibited similar model fit (see S2 Fig for detailed information). Because interpretable conclusions about the comparison between the Bookmark procedure and cMRM could be threatened by shifting item difficulty parameter locations, we further correlated the item parameters of the respective Rasch model against the final cMRM. For all samples the item difficulty parameters did not differ between the respective Rasch model and cMRM (*r* = 1.00). The item difficulty parameters of the reading items from the Rasch models and final cMRMs are given in S9 and S10 Tables and in S3 Fig.

For each sample, cMRMs with one to seven classes were fitted to the data. All the models were estimated using maximum likelihood with heteroscedasticity-robust standard errors [52]. To prevent local solutions [53], each model was estimated with 500 random start values and 50 optimizations. Different criteria were used to determine the best fitting model and evaluate the robustness of the chosen class solution [34–36]: First, the Akaike information criterion (AIC; [54]), Bayesian information criterion (BIC; [55]), and sample-adjusted Bayesian information criterion (aBIC; [56]) served as quality measures of the statistical models, with lower values indicating a better fit [35, 57]. Moreover, the Vuong–Lo–Mendell–Rubin likelihood ratio test (VLMR-LRT; [58]) and the bootstrapped likelihood ratio test (BLRT) represent statistical tests that indicate whether adding an additional class significantly improves the model fit. A significant difference indicates that the model with k latent classes provides a better fit to the data as compared to the model with k-1 latent classes [35, 59]. Given the large sample sizes in our study, the Type 1 error was set at α = .001. In case the criteria preferred different class solutions, more weight was given to the aBIC and the BLRT, because they proved to be the most robust indicators of model fit in mixture modeling [35, 60, 61]. Second, as indicators of classification quality, we also considered the relative entropy and average posterior classification probabilities (ACPs) for the most likely class membership. The relative entropy could range from 0 to 1, with values greater than .80 being considered as high class discrimination of the latent classes, greater than .60 as medium, and greater than .40 as low [62]. According to Nagin [63], an average posterior probability greater than .70 may be considered acceptable and a probability below .70 as problematic. Third, as there is no generally accepted rule of thumb for a minimum class size, we decided that the classes should include at least 5% of the sample [64]. Otherwise, a class was deemed not important and unlikely to replicate across different (particularly smaller) samples. Fourth, the class solutions were further evaluated in terms of their replication success across different independent samples. The two adult samples were each considered as an independent replication for the same population. In contrast, for the student sample a split-half procedure was applied that evaluated the class solutions in two random splits (without replacement) of the sample (*n*_1_ = 6,949, *n*_2_ = 6,948). The two split-half samples did not differ significantly on gender, migration background, age, or cognitive abilities (i.e., reading competence, receptive vocabulary, reasoning abilities).

### Agreement between the standard-setting procedures

The agreement between the Bookmark procedure and cMRM in the assignment of individuals to either the low-literacy or literacy group was examined by focusing on only two classes identified by the cMRM: the class with the lowest reading proficiency represented the low-literacy group, whereas all remaining individuals were pooled into the literacy group.

A 2 × 2 contingency table (see Table 1) was created to calculate the following five measures to tap different facets of classification agreement: McNemar’s χ2 test, Cohen’s Kappa κ, sensitivity, specificity and a within-proficiency disagreement rate (see Table 2 for a definition of these measures).

**Table 1:** Contingency table for agreement between the bookmark procedure (BM) and constrained Mixture Rasch Model (cMRM).

	BM low-literate	BM literate	
cMRM low-literate	A	B	A+B
cMRM literate	C	D	C+D
	A+C	B+D	A+B+C+D

**Table 2:** Agreement measures.

Agreement measures	Formula	Definition
McNemar’s χ2 test	(B–C)^2^ / (B + C)	Test for comparable marginal proportions (i.e., similar rates of literate readers)
Cohen’s Kappa κ	*(p*_*0*_ *—p*_*e)*_ */(1- p*_*e*_ *)*	Proportion of agreement on both the agreement and disagreement in proficiency assignment, corrected for change agreement (Global agreement)
	= 2*(A*D–C*B) / [(A+B) * (B+D)+(A+C)*(C+D)]	
Sensitivity (true-positive rate)	A/(A + C)	Proportion of readers correctly identified as low-literate readers with the cMRM as detected by the Bookmark procedure
Specificity (true-negative rate)	D/(D + B)	Proportion of readers correctly identified as literate readers with the cMRM as detected by the Bookmark procedure
Disagreement rate for low-literacy assignment (DIS_low)_	(B+C)/(A+B+C)	Proportion of individuals who were classified as low-literate within the Bookmark procedure but literate within the cMRM and vice versa, relative to the proportion of individuals who were classified as low-literate within both procedures
Disagreement rate for literacy assignment (DIS_lit_)	(B+C)/(B+C+D)	Proportion of individuals who were classified as literate within the Bookmark procedure but low-literate within the cMRM and vice versa, relative to the proportion of individuals who were classified as literate within both procedures

#### McNemar’s χ^2^ test

McNemar’s χ2 test [65] was used to examine if the proportion of literate readers were present in the same proportions in the Bookmark procedure and cMRM.

#### Cohen’s Kappa κ

Cohen’s Kappa κ measures the global agreement between the Bookmark procedure and cMRM [66]. According to Landis and Koch [67], agreement is poor if Cohen’s κ is less than .20, fair if Cohen’s κ is between .21 and .40, moderate if Cohen’s κ is between .41 and .60, substantial if Cohen’s κ is between .61 and .80, almost perfect if Cohen’s κ is between .81 and .99, and perfect if Cohen’s κ equals 1.00. The level of significance was set at 1%.

#### Sensitivity and specificity

Sensitivity and specificity are the most commonly used measures of the accuracy of a diagnostic test as compared to an existing reference test (gold standard) [68, 69]. The sensitivity of a test refers to the proportion of individuals correctly identified with a condition (here: low literacy) as detected by the gold standard. Specificity, in contrast, is defined as the proportion of individuals correctly identified without a condition (here: literacy) as detected by the gold standard [68, 69]. The Bookmark procedure was defined as the reference standard-setting procedure (gold standard). However, it is important to note that sensitivity and specificity of the “new” standard-setting procedure (here: cMRM) is influenced by sensitivity and specificity of the gold standard itself. The gold standard reference is considered to be true–by definition its sensitivity and specificity should be perfect. But, like any standard-setting procedure, the Bookmark procedure is imperfect, since it is vulnerable to interpretation and measurement challenges [1–6].

#### Within-proficiency level disagreement rate

To overcome the challenge of defining a gold standard, when there is no perfect standard-setting procedure, we calculated an alternative measure for assessing agreement between both procedures, a within-proficiency level disagreement rate. It measures the proportion of agreement within the low-literacy proficiency assignment (DIS_low_) and within the literacy assignment (DIS_lit_).

## Results

The results are divided into three subsections. First, we describe the determination of the number of latent classes in the cMRMs for each sample. Then, the best-fitting class solution is presented. Finally, agreement between the Bookmark procedure and cMRM in the assignment of individuals to either the low-literacy or literacy group is evaluated.

### Determination of the number of latent classes

#### Student sample

**Fig 1:**
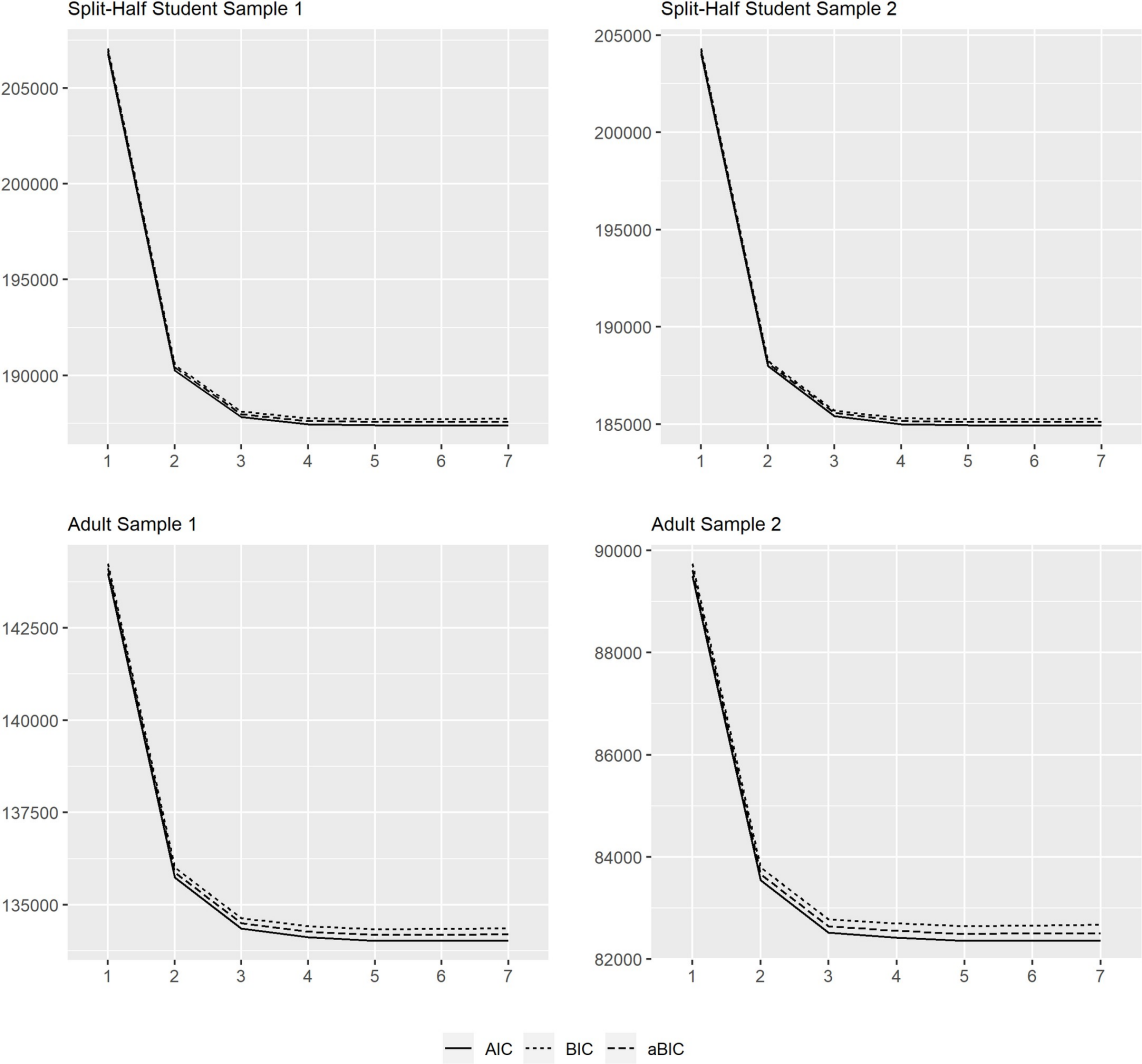
Scree plots for information criteria in different samples.

Considering the information criteria, the cMRMs for both samples resulted in the lowest AIC, BIC, and aBIC values for the 6-class solution. As can be seen in the scree plot (Fig 1), the relative decline is quite small in the AIC, BIC, and aBIC values after the 3-class solution. Considering the likelihood-based tests, the VLMR test was significant up to the 5-class-solution, and the BLRT confirmed that each model had a statistically better fit than the preceding one. VLMR and BLRT were not available for the 7-class solution due to convergence problems (see S1 and S2 Tables for detailed information).

However, the proportion of students within a class fell below our 5% criterion from the 5-class solution onward in both the split-half samples (see S3 and S4 Tables for detailed information). In addition, from the 5-class solution onward there were increasing deviations in the proportion of students within a class. These results speak in favor of the 4-class solution. As the 4-class solution for the two split-half samples also demonstrated an acceptable classification quality, the 4-class-solution was chosen for the comparison with the Bookmark procedure.

#### Adult samples

Considering the information criteria, the cMRMs for both adult samples resulted in the lowest BIC and aBIC values for the 5-class solution, while the 6-class solution yielded the best AIC in Adult Sample 1 and the 5-class solution yielded the best AIC in Adult Sample 2 (see S5 and S6 Tables for detailed information). As can be seen in Fig 1, one observes a significant decrease in AIC, BIC, and aBIC values up to about the 3-class solution and a much slower decline until the 7-class solution. Considering the likelihood-based tests, the VLMR test and BLRT test were significant up to the 5-class-solution, suggesting the best fit for the 5-class-solution. However, in both samples the proportion of adults within a class fell below our 5% criterion from the 4-class solution onward (S7 and S8 Tables for detailed information). Thus, the 3-class solution was considered the most accurate for both samples. The classification quality of the 3-class solution was also acceptable for both adult samples. First, the entropy values were medium at .70 and .72, respectively. Second, the class assignment probabilities with ACPs larger than .80 indicated a relatively high certainty in class assignment.

### Description of the constrained mixture Rasch models

**Fig 2:**
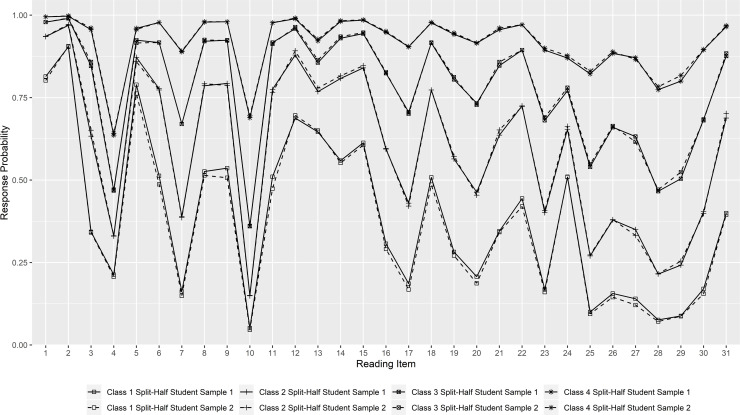
Response probability profile of “correct answer” for 4-class solution among the split-half student samples.

**Fig 3:**
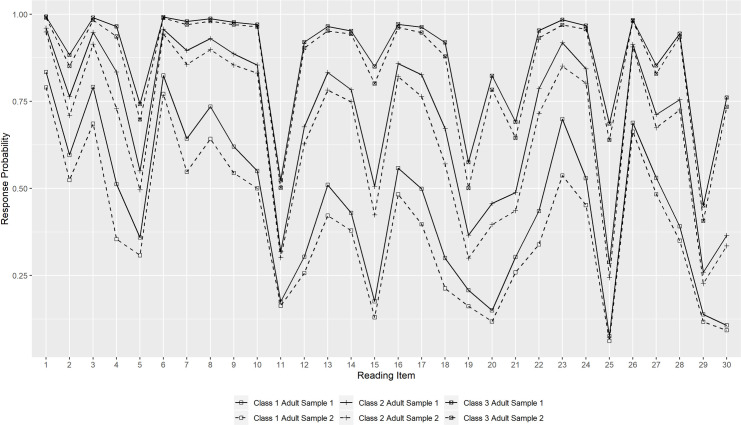
Response probability profile of “correct answer” for 3-class solution among the adult samples.

**Fig 4:**
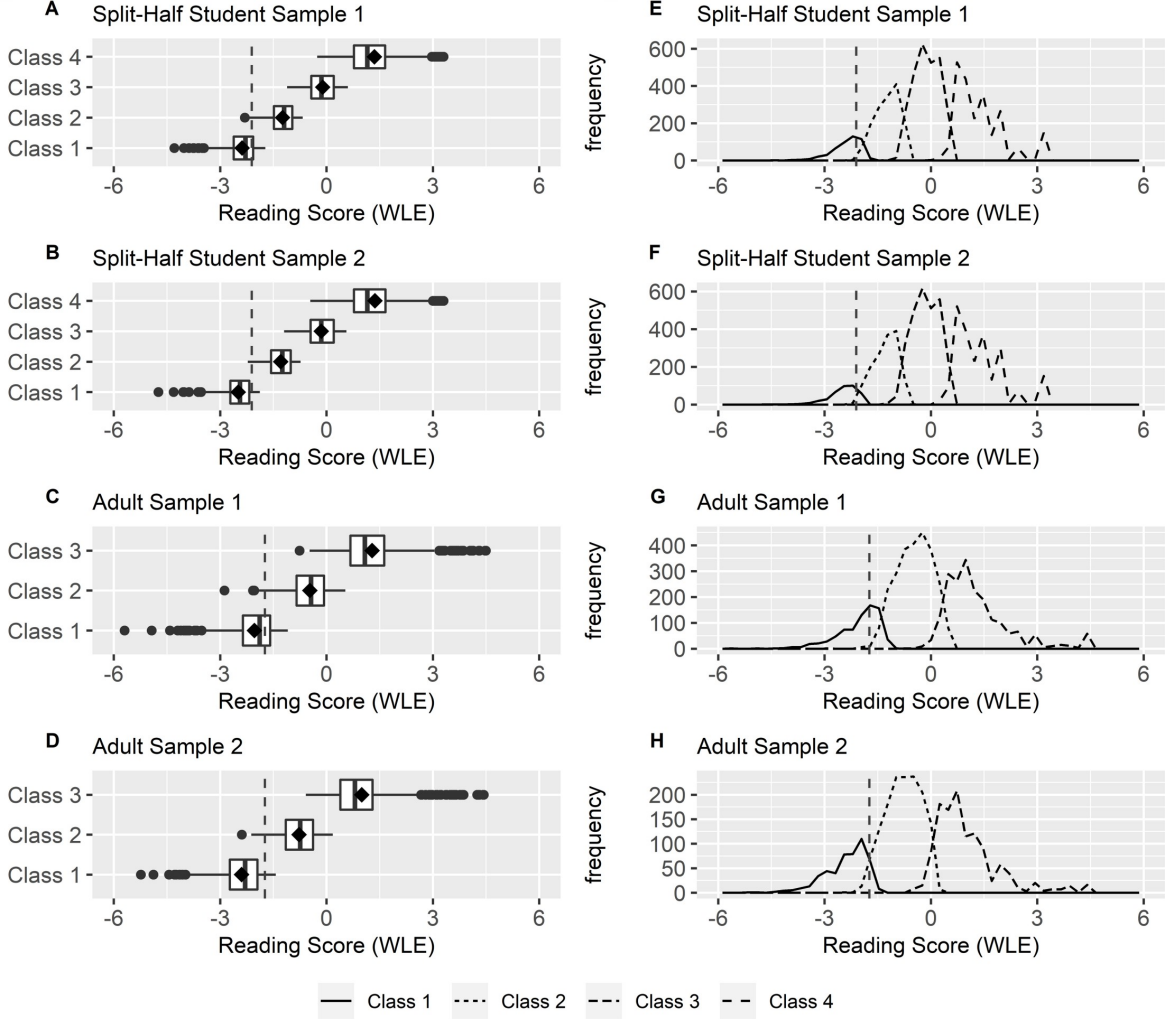
Distributions of the latent classes on reading score (WLE) in different samples. Boxplots in Fig 4A to 4D display the class-specific reading competence distribution for the different samples. Frequency polygons in Fig 4E to 4H display the class-specific reading ability distribution and frequency for the different samples. The dashed vertical lines mark the cut scores for the Bookmark procedure.

Figs 2 and 3 present the class-specific response probabilities for each reading item of the two split-half student samples and the two adult samples. For dichotomous items, the response probability indicates the probability of a respondent to choose the correct response option, while for polytomous ‘super-items’ the highest category was chosen. For example, the response probability for item 4 in the two split-half student samples indicates the probability of answering both of the two subtasks correctly. Class 1 has in all samples the lowest response probabilities for all items and is therefore in the following referred to as the “low literacy group”. With increasing class, the response probabilities for each item also increase. This is also reflected in the distribution of reading competence across the classes (Fig 4), which shows that the latent classes represent quantitative differences in the respondents’ reading competence (see also S4 and S5 Figs for the association between class membership and the WLE estimates). As can be seen, the response profiles of the respective classes for the two split-half student samples run almost parallel, and the same applies to the distribution of reading competence across the classes. The response probability for each item is lower for Adult Sample 2 than for Adult Sample 1, which is also reflected in a lower mean reading competence of each class for Adult Sample 2 (Fig 4). The respective descriptive statistics are also summarized in S11 and S12 Tables.

### Association between the bookmark procedure and constrained mixture Rasch model

The percentages of individuals within the low-literacy group for all samples are presented in Table 3. As can be seen, the student sample is no longer considered separately in the following analysis, as it represents one population. For the student sample and Adult Sample 2, there were only small differences in the percentage of low-literate readers between the Bookmark procedure and cMRM. For Adult Sample 1, there were larger differences in the percentage of low-literate readers between the Bookmark procedure and cMRM, affecting about 6% of the sample. For all samples, the Bookmark procedure yielded a lower percentage of individuals who fell into the lowest proficiency level than did the cMRM. The proportion of low-literate readers was 4.95%, 8.91%, and 15.39% for the student sample, Adult Sample 1, and Adult Sample 2, respectively, when the Bookmark procedure was applied, and 6.36%, 14.45% and 15.64% for the respective cMRMs. These differences in proportions were also significant (*p* < .001) following respective McNemar’s χ^2^ tests for the student sample and Adult Sample 1, while there was no significant differences for Adult Sample 2 (see Table 5).

**Table 3:** Percentage of individuals within the low-literacy group.

	Student Sample	Adult Sample 1	Adult Sample 2
Bookmark procedure	4.95	8.91	15.39
Constrained mixture Rasch model	6.36	14.45	15.64

The relationship between the individuals who were assigned to the low-literacy group or literacy group detected by the Bookmark procedure and those who were assigned to the low-literacy group or literacy group detected by the cMRM (contingency) is shown in Table 4.

**Table 4:** Contingency table for agreement between the bookmark procedure (BM) and constrained Mixture Rasch Model (cMRM) in different samples.

Student Sample			
	BM low-literate	BM literate	Total
cMRM low-literate	675	209	884
	(4.86%)	(1.50%)	(6.36%)
cMRM literate	13	13000	13013
	(0.09%)	(93.55%)	(93.64%)
Total	688	13209	13897
	(4.95%)	(95.05%)	(100.00%)
Adult Sample 1			
	BM low-literate	BM literate	Total
cMRM low-literate	464	306	770
	(8.71%)	(5.74%)	(14.45%)
cMRM literate	11	4549	4560
	(0.21%)	(85.34%)	(85.55%)
Total	475	4855	5330
	(8.92%)	(91.08%)	(100.00%)
Adult Sample 2			
	BM low-literate	BM literate	Total
cMRM low-literate	457	35	492
	(14.53%)	(1.11%)	(15.64%)
cMRM literate	27	2626	2653
	(0.86%)	(83.50%)	(84.36%)
Total	484	2661	3145
	(15.39%)	(84.61%)	(100.00%)

Table 5 displays the respective agreement measures. The Kappa *κ* coefficient of consistency was almost perfect at .85 and .93 for the student sample and Adult Sample 2, respectively, but relatively lower, albeit still substantial, at .71 for Adult Sample 1. For all samples, the cMRMs showed high sensitivity and specificity for detecting low-literate and literate readers, respectively, ranging between 94.42% and 98.11% sensitivity and 93.70% to 98.68% specificity. Furthermore, the disagreement rate showed that for students, the Bookmark procedure and cMRM disagreed by 24.75% for the low-literacy group and by 1.68% for the literacy group. For Adult Sample 2, the Bookmark procedure and cMRM disagreed by 11.95% for the low-literacy group and by 2.31% for the literacy group. For Adult Sample 1, the disagreement rate for the low-literacy group was 40.59% and 6.51% for the literacy group (see Table 5).

**Table 5:** Measures for agreement between the bookmark procedure (BM) and constrained Mixture Rasch Model (cMRM) in different samples.

	McNemar’s χ^2^		Kappa κ			Sensi-tivity	Speci-ficity	DIS_low_	DIS_lit_
	*χ*^2^ (1)	*p*	κ (*SE*)	[95% CI]	*p*	%	%	%	%
Student Sample	171	< .001	.85 (.010)	[.83,.87]	< .001	98.11	98.42	24.75	1.68
Adult Sample 1	273	< .001	.71 (.015)	[.68,.74]	< .001	97.68	93.70	40.59	6.51
Adult Sample 2	0.79	.374	.93 (.009)	[.91,.94]	< .001	94.42	98.68	11.95	2.31

95% CI = 95% confidence interval, DIS_low_ = Disagreement rate for low-literacy assignment, DIS_lit_ = Disagreement rate for literacy assignment.

## Discussion

In this study, we compared the results of two different standard-setting procedures that have so far been rarely used in the context of external validity research—the Bookmark procedure (judgement-based approach) and constrained mixture Rasch modeling (cMRM) (model-based approach). The comparison was conducted with three subsamples within the German National Educational Panel Study.

Overall, the results showed high consistency in the proficiency level assignment in the student sample and Adult Sample 2, whereas more pronounced differences were observed in Adult Sample 1. The assignment to the respective proficiency levels in the student sample and Adult Sample 2 differed by approximately 2% between the two standard-setting procedures. Thus, neither procedure was significantly different, suggesting that both the Bookmark procedure and cMRM provided feasible cut scores for differentiating low-literate and literate readers, and therefore both can be used for further analysis. These results are quite notable, as both procedures differ substantially in their assumptions of the underlying data structures. In the Bookmark procedure, the calculation of the item difficulty parameters is based on item response theory (IRT) assuming a normal trait distribution of all persons on the trait continuum, whereas MRM assumes that the trait distribution is a mixture of normal distributions in latent classes [31–33]. However, we used a constrained version of MRM that specified the normal distribution of the latent proficiency as a set of (ordered) qualitatively different classes with different means but no within-class variability. Thus, the variance of the Rasch model is indirectly represented by the spread of the means in the different latent classes of the cMRM. Thus, the Rasch model can be considered a more general case of the cMRM. We might have expected a pronounced bias between the two procedures if the cMRM exhibited a substantial worse fit. However, the constraints resulted in a negligible change in fit. This can also be seen from the high correlation of the item difficulty parameters.

In light of prior discussions of the importance of using different standard-setting approaches for proficiency interpretation, the degree of inconsistency in classification was further not as pronounced as might have been expected. It might be argued that the high degree of consistency reflects the development of low literacy over the life course as the result of an unfavorable socialization in literacy that leaves behind a small and unique group of low-literate readers whose reading proficiency developed very slowly or perhaps even stopped and declined [70, 71]. This may explain why the Bookmark procedure and cMRM were able to detect and characterize this unique group of readers at the lower end of the reading trait distribution.

Our results further showed that the choice of classification indicators is an important factor to consider when interpreting validity in assigning individuals to different levels of proficiency. For example, the results showed that sensitivity and specificity of the respective cMRMs were surprisingly high. But, as outlined above, sensitivity and specificity are somewhat problematic indicators because both assume the Bookmark procedure as the gold standard and, therefore, the results should not be overestimated. Our proposed indicator, the within-proficiency disagreement rate, provides an alternative, when no perfect gold standard exist, and has the distinct advantage of showing how two standard-setting procedures disagree in assigning individuals to a particular proficiency level.

With regard to Adult Sample 1, the comparison yielded some larger discrepancies in the cut scores between the two standard-setting procedures that affected about 6% of the sample and occurred especially at the low proficiency level. The results indicate that the ability distribution may be a reason for the lower agreement. Although both adult samples covered a wide range of ability distribution, Adult Sample 2 had an overall lower reading competence and participants with lower abilities were more strongly represented in the low ability regions than were participants of Adult Sample 1. Therefore, Adult Sample 1 may not have provided the necessary differentiation in the low ability regions, which may have led to an overestimation of the “true” low reading proficiency level. The implication of this finding is that the cut score placement is sensitive to the variance of the sample’s ability distribution when using a cMRM. This underlines the need to ensure that the sample has sufficient variance when using a cMRM for setting standards. It is less likely that meaningful interpretations of the proficiency levels can be made if potential ability levels are excluded from or underrepresented in the range of possible cut scores. It is further possible that the larger discrepancies in Adult Sample 1 could be a result of a generally worse model fit of both, the Rasch model and cMRM. Therefore, further studies examining the influence of model fit on standard setting would be of interest.

Moreover, in line with previous results, the comparison showed that the cut scores determined by the Bookmark procedure are lower than those resulting from other standard-setting procedures [9, 11–15]. The differences between the two standard-setting procedures become relevant when the standards are used for research purposes or for interventions. For example, if the focus is on the stability and changeability of low reading proficiency, a stricter cut score is probably more appropriate. If, on the other hand, the cut scores serve the purpose of allocation to an intervention, the cMRM provides liberal cut scores.

Another main implication is that the use of two different standard-setting procedures provides a promising alternative for cross-validation. On the one hand, the cMRM does not rely on external panelists’ judgements, which makes the method not only more objective but also less resource-intensive in comparison to other judgement-based procedures. Thus, it provides a promising alternative to previous validation studies. On the other hand, given the satisfactory agreement between the two procedures, the Bookmark procedure provides the necessary content validity, which the cMRM does not, and thus allows a criterion-referenced interpretation of a person’s skills at a given proficiency level.

There are several limitations to this study, which will need to be addressed in future studies. First, the focus here was not to examine the effect of the ability distribution on estimating proficiency levels within the cMRM. In further research, it may be worth examining this relationship. The second limitation of this study is that only one cut score was provided for the Bookmark procedure, and, therefore, the proficiency levels had to be pooled from class 2 upwards in the cMRM. It would certainly have enhanced the significance of the comparison if multiple cut scores had been set for the Bookmark procedure as well. Thus, in future studies, comparison with multiple cut scores should be conducted to support generalizability of the results. And finally, a challenge with this sort of analysis is how reading proficiency was modeled by the test developers. Reading competence was modelled as a continuous trait, thus, the instruments were not designed to capture distinct well-defined proficiency groups. Although our findings support the validity of the proficiency levels, it is more difficult to have a clear signal in the data to detect latent proficiency classes when the instrument aims to capture a continuous trait. Therefore, further studies would be interesting to validate both approaches on instruments explicitly designed to capture different proficiency levels.

## Supporting information

S1 Table Fit indices and classification quality for model specifications in the first split-half student sample. (DOCX)

S2 Table Fit indices and classification quality for model specifications in the second split-half student sample. (DOCX)

S3 Table Class proportions in the first split-half student sample. (DOCX)

S4 Table Class proportions in the second split-half student sample. (DOCX)

S5 Table Fit indices and classification quality for model specifications in the first adult sample. (DOCX)

S6 Table Fit indices and classification quality for model specifications in the second adult sample. (DOCX)

S7 Table Class proportions in the first adult sample. (DOCX)

S8 Table Class proportions in the second adult sample. (DOCX)

S9 Table Item parameters among the split-half student samples. (DOCX)

S10 Table Item parameters among the adult samples. (DOCX)

S11 Table Descriptive statistics by latent classes on reading competence among the split-half student samples. (DOCX)

S12 Table Descriptive statistics by latent classes on reading competence among the adult samples. (DOCX)

S1 Fig Distribution of reading competence (WLE) by sample. The density plots display the reading ability distribution for the different samples according to the respective Rasch models. The dashed vertical lines mark the cut scores for the Bookmark procedure.
(TIF)

S2 Fig Univariate and bivariate item fit statistics by sample. The figure shows the univariate and bivariate model fit information for the respective Rasch models and final cMRM class solutions among the two split-half student samples and two adult samples. The univariate model fit compared predicted and observed frequencies of responses for all reading items marginally. The bivariate model fits compared predicted and observed frequencies of responses for each pair of reading items. Given the large sample size, standardized residuals > |6| were considered as having a noticeable item misfit. Both model approaches, the Rasch model and cMRM showed a comparable model fit and therefore indicate that both approaches represent an acceptable measurement model. For univariate model fit, 0.00% to 2.90% items showed a noticeable misfit. For bivariate model fit, 1.79% to 5.02% items showed a noticeable misfit. Note: Some local dependence is induced by the testlet design in NEPS, that is, that the reading tests consisted of five texts, each with a set of reading items referring to the same stimulus, and by specific response formats that consist of item bundles referring to a common stimulus.
(TIF)

S3 Fig Item difficulty parameter of the respective Rasch model and cMRM by sample. (TIF)

S4 Fig Distribution of the most likely class membership depending on the WLE estimates of the person parameters by the split-half student samples. The figure shows the distribution of the most likely class membership depending on the WLE estimates of the person parameters by the two split-half student sample. As can be seen, there is a clear association between class membership and the WLE estimates; less competent persons are primarily belong to class 1 (here: low literacy group).
(TIF)

S5 Fig Distribution of the most likely class membership depending on the WLE estimates of the person parameters by the adult samples. The figure shows the distribution of the most likely class membership depending on the WLE estimates of the person parameters by the two adult sample. As can be seen, there is a clear association between class membership and the WLE estimates; less competent persons are primarily belong to class 1 (here: low literacy group).
(TIF)
