# Supplementary material for: Setting a standard for low reading proficiency: A comparison of the bookmark procedure and constrained mixture Rasch model
Source: PLoS One. 2021 Nov 29;16(11):e0257871. doi: 10.1371/journal.pone.0257871 (PMC8629253; doi:10.1371/journal.pone.0257871)
Supplement: S1 Table — (DOCX) [file pone.0257871.s001.docx]

**S1 Table. Fit indices and classification quality for model specifications in the first split-half student sample.**

| Model | 1-class | 2-classes | 3-classes | 4-classes | 5-classes | 6-classes | 7-classes |
| --- | --- | --- | --- | --- | --- | --- | --- |
| Parameters | 38 | 40 | 42 | 44 | 46 | 48 | 50 |
| AIC | 206819 | 190264 | 187827 | 187449 | 187405 | 187384 | 187386 |
| BIC | 207079 | 190537 | 188114 | 187751 | 187720 | 187712 | 187728 |
| aBIC | 206958 | 190410 | 187981 | 187611 | 187573 | 187560 | 187569 |
| VLMR | n/a | <.001 | <.001 | <.001 | <.001 | .002 | n/a |
| BLRT | n/a | <.001 | <.001 | <.001 | <.001 | <.001 | n/a |
| Entropy | n/a | .83 | .75 | .68 | .64 | .67 | .61 |
| Range of ACPs | n/a | .94–.96 | .86–.90 | .79–.86 | .73–.80 | .61–.78 | .58–.75 |

Parameters = number of model parameters; AIC = Akaike information criterion; BIC = Bayesian information criterion; aBIC = Bayesian information criterion adjusted to the sample size; VLMR = Vuong–Lo–Mendell–Rubin likelihood ratio test; BLRT = bootstrapped likelihood ratio test; ACP = Average latent class probabilities for most likely latent class membership by latent class. VLMR and BLRT were not available for 7-classes solution due to convergence problems.
