# Supplementary material for: Setting a standard for low reading proficiency: A comparison of the bookmark procedure and constrained mixture Rasch model
Source: PLoS One. 2021 Nov 29;16(11):e0257871. doi: 10.1371/journal.pone.0257871 (PMC8629253; doi:10.1371/journal.pone.0257871)
Supplement: S2 Table — (DOCX) [file pone.0257871.s002.docx]

**S2 Table. Fit indices for model specifications in the second split-half student sample.**

| Model | 1-class | 2-classes | 3-classes | 4-classes | 5-classes | 6-classes | 7-classes |
| --- | --- | --- | --- | --- | --- | --- | --- |
| Parameters | 38 | 40 | 42 | 44 | 46 | 48 | 50 |
| AIC | 204056 | 187976 | 185416 | 185001 | 184942 | 184928 | 184930 |
| BIC | 204316 | 188250 | 185704 | 185302 | 185257 | 185257 | 185272 |
| aBIC | 204195 | 188123 | 185570 | 185163 | 185111 | 185104 | 185113 |
| VLMR | n/a | <.001 | <.001 | <.001 | <.001 | .009 | n/a |
| BLRT | n/a | <.001 | <.001 | <.001 | <.001 | <.001 | n/a |
| Entropy | n/a | .82 | .76 | .69 | .63 | .66 | .65 |
| Range of ACPs | n/a | .93–.95 | .85–.92 | .78–.86 | .69–.84 | .68–.82 | .65–.82 |

Parameters = number of model parameters; AIC = Akaike information criterion; BIC = Bayesian information criterion; aBIC = Bayesian information criterion adjusted to the sample size; VLMR = Vuong–Lo–Mendell–Rubin likelihood ratio test; BLRT = bootstrapped likelihood ratio test; ACP = Average latent class probabilities for most likely latent class membership by latent class. VLMR and BLRT were not available for 7-classes solution due to convergence problems.
