# Supplementary material for: Setting a standard for low reading proficiency: A comparison of the bookmark procedure and constrained mixture Rasch model
Source: PLoS One. 2021 Nov 29;16(11):e0257871. doi: 10.1371/journal.pone.0257871 (PMC8629253; doi:10.1371/journal.pone.0257871)
Supplement: S3 Table — (DOCX) [file pone.0257871.s003.docx]

**S3 Table. Class proportions in the first split-half student sample.**

| Model | Class 1  in % | Class 2  in % | Class 3  in % | Class 4  in % | Class 5  in % | Class 6  in % | Class 7  in % |
| --- | --- | --- | --- | --- | --- | --- | --- |
| 1-class | 100.00  (100.00) |  |  |  |  |  |  |
| 2-classes | 34.68  (34.09) | 65.32  (65.91) |  |  |  |  |  |
| 3-classes | 15.04  (14.48) | 41.69  (41.52) | 43.28  (44.00) |  |  |  |  |
| 4-classes | 7.58  (6.99) | 22.23  (21.70) | 39.02  (38.54) | 31.17  (32.78) |  |  |  |
| 5-classes | 2.86  (2.33) | 11.40  (10.89) | 25.25  (24.49) | 35.71  (37.06) | 24.79  (25.22) |  |  |
| 6-classes | 1.35  (0.92) | 8.83  (8.55) | 20.32  (19.73) | 33.85  (34.25) | 32.50  (34.45) | 3.15  (2.10) |  |
| 7-classes | 0.94  (0.00) | 7.52  (7.08) | 15.78  (15.80) | 23.32  (23.15) | 26.22  (25.08) | 25.31  (28.23) | 0.91  (0.65) |

Class proportions based on the estimated posterior probabilities (most likely latent class membership in brackets). Classes ordered by mean of class.
