# Supplementary material for: Setting a standard for low reading proficiency: A comparison of the bookmark procedure and constrained mixture Rasch model
Source: PLoS One. 2021 Nov 29;16(11):e0257871. doi: 10.1371/journal.pone.0257871 (PMC8629253; doi:10.1371/journal.pone.0257871)
Supplement: S4 Table — (DOCX) [file pone.0257871.s004.docx]

**S4 Table. Class proportions in the second split-half student sample.**

| Model | Class 1  in % | Class 2  in % | Class 3  in % | Class 4  in % | Class 5  in % | Class 6  in % | Class 7  in % |
| --- | --- | --- | --- | --- | --- | --- | --- |
| 1-class | 100.00  (100.00) |  |  |  |  |  |  |
| 2-classes | 35.90  (35.39) | 64.10  (64.61) |  |  |  |  |  |
| 3-classes | 13.17  (12.49) | 42.46  (42.06) | 44.37  (45.45) |  |  |  |  |
| 4-classes | 6.08  (5.60) | 21.74  (21.04) | 39.23  (40.03) | 32.96  (33.33) |  |  |  |
| 5-classes | 4.91  (4.55) | 16.79  (15.99) | 32.97  (33.15) | 33.93  (36.72) | 11.41  (9.60) |  |  |
| 6-classes | 0.27  (0.19) | 5.69  (5.27) | 17.52  (16.70) | 32.84  (33.00) | 33.64  (36.87) | 10.04  (7.97) |  |
| 7-classes | 0.26  (0.17) | 5.61  (5.11) | 17.07  (16.55) | 31.62  (32.11) | 29.86  (31.16) | 14.97  (14.90) | 0.61  (0.00) |

Class proportions based on the estimated posterior probabilities (most likely latent class membership in brackets). Classes ordered by mean of class.
