# Supplementary material for: Setting a standard for low reading proficiency: A comparison of the bookmark procedure and constrained mixture Rasch model
Source: PLoS One. 2021 Nov 29;16(11):e0257871. doi: 10.1371/journal.pone.0257871 (PMC8629253; doi:10.1371/journal.pone.0257871)
Supplement: S5 Table — (DOCX) [file pone.0257871.s005.docx]

**S5 Table. Fit indices and classification quality for model specifications in the first adult sample.**

| Model | 1-class | 2-classes | 3-classes | 4-classes | 5-classes | 6-classes | 7-classes |
| --- | --- | --- | --- | --- | --- | --- | --- |
| Parameters | 39 | 41 | 43 | 45 | 47 | 49 | 51 |
| AIC | 143977 | 135738 | 134359 | 134122 | 134023 | 134022 | 134024 |
| BIC | 144234 | 136008 | 134642 | 134419 | 134332 | 134344 | 134360 |
| aBIC | 144110 | 135877 | 134505 | 134276 | 134183 | 134189 | 134198 |
| VLMR | n/a | <.001 | <.001 | <.001 | <.001 | .110 | .094 |
| BLRT | n/a | <.001 | <.001 | <.001 | <.001 | .040 | .143 |
| Entropy | n/a | .77 | .70 | .68 | .70 | .72 | .70 |
| Range of ACPs | n/a | .99–.94 | .84–.87 | .79–.83 | .77–.82 | .69–.81 | .52–.76 |

Parameters = number of model parameters; AIC = Akaike information criterion; BIC = Bayesian information criterion; aBIC = Bayesian information criterion adjusted to the sample size; VLMR = Vuong–Lo–Mendell–Rubin likelihood ratio test; BLRT = bootstrapped likelihood ratio test; ACP = Average latent class probabilities for most likely latent class membership by latent class.
