# Supplementary material for: Setting a standard for low reading proficiency: A comparison of the bookmark procedure and constrained mixture Rasch model
Source: PLoS One. 2021 Nov 29;16(11):e0257871. doi: 10.1371/journal.pone.0257871 (PMC8629253; doi:10.1371/journal.pone.0257871)
Supplement: S6 Table — (DOCX) [file pone.0257871.s006.docx]

**S6 Table. Fit indices and classification quality for model specifications in the second adult sample.**

| Model | 1-class | 2-classes | 3-classes | 4-classes | 5-classes | 6-classes | 7-classes |
| --- | --- | --- | --- | --- | --- | --- | --- |
| Parameters | 39 | 41 | 43 | 45 | 47 | 49 | 51 |
| AIC | 89500 | 83546 | 82522 | 82422 | 82356 | 82357 | 82358 |
| BIC | 89736 | 83794 | 82782 | 82695 | 82640 | 82653 | 82667 |
| aBIC | 89612 | 83664 | 82646 | 82552 | 82491 | 82498 | 82505 |
| VLMR | n/a | <.001 | <.001 | .004 | <.001 | .070 | .167 |
| BLRT | n/a | <.001 | <.001 | <.001 | <.001 | .130 | .109 |
| Entropy | n/a | .78 | .72 | .74 | .71 | .73 | .66 |
| Range of ACPs | n/a | .92–.95 | .85–.89 | .79–.88 | .74–.84 | .72–.84 | .56–.77 |

Parameters = number of model parameters; AIC = Akaike information criterion; BIC = Bayesian information criterion; aBIC = Bayesian information criterion adjusted to the sample size; VLMR = Vuong–Lo–Mendell–Rubin likelihood ratio test; BLRT = bootstrapped likelihood ratio test; ACP = Average latent class probabilities for most likely latent class membership by latent class.
