# Supplementary material for: Setting a standard for low reading proficiency: A comparison of the bookmark procedure and constrained mixture Rasch model
Source: PLoS One. 2021 Nov 29;16(11):e0257871. doi: 10.1371/journal.pone.0257871 (PMC8629253; doi:10.1371/journal.pone.0257871)
Supplement: S7 Table — (DOCX) [file pone.0257871.s007.docx]

**S7 Table. Class proportions in the first adult sample.**

| Model | Class 1  in % | Class 2  in % | Class 3  in % | Class 4  in % | Class 5  in % | Class 6  in % | Class 7  in % |
| --- | --- | --- | --- | --- | --- | --- | --- |
| 1-class | 100.00  (100.00) |  |  |  |  |  |  |
| 2-classes | 35.01  (34.34) | 64.99  (65.66) |  |  |  |  |  |
| 3-classes | 15.21  (14.45) | 47.33  (48.34) | 37.46  (37.21) |  |  |  |  |
| 4-classes | 3.95  (3.37) | 20.69  (19.87) | 46.14  (47.78) | 29.22  (28.99) |  |  |  |
| 5-classes | 3.42  (2.94) | 18.12  (17.15) | 41.68  (43.21) | 34.39  (34.88) | 2.40  (1.82) |  |  |
| 6-classes | 0.30  (0.13) | 3.77  (3.19) | 18.19  (17.40) | 41.43  (43.19) | 33.96  (34.28) | 2.35  (1.82) |  |
| 7-classes | 0.26  (0.11) | 3.66  (3.13) | 17.70  (16.87) | 40.03  (41.65) | 33.41  (35.56) | 3.75  (0.90) | 1.20  (1.78) |

Class proportions based on the estimated posterior probabilities (most likely latent class membership in brackets). Classes ordered by mean of class.
