# Supplementary material for: Setting a standard for low reading proficiency: A comparison of the bookmark procedure and constrained mixture Rasch model
Source: PLoS One. 2021 Nov 29;16(11):e0257871. doi: 10.1371/journal.pone.0257871 (PMC8629253; doi:10.1371/journal.pone.0257871)
Supplement: S8 Table — (DOCX) [file pone.0257871.s008.docx]

**S8 Table. Class proportions in the second adult sample.**

| Model | Class 1  in % | Class 2  in % | Class 3  in % | Class 4  in % | Class 5  in % | Class 6  in % | Class 7  in % |
| --- | --- | --- | --- | --- | --- | --- | --- |
| 1-class | 100.00  (100.00) |  |  |  |  |  |  |
| 2-classes | 37.28  (37.01) | 62.72  (62.99) |  |  |  |  |  |
| 3-classes | 16.34  (15.64) | 45.43  (46.36) | 38.23  (38.00) |  |  |  |  |
| 4-classes | 14.80  (14.02) | 40.96  (41.30) | 40.51  (41.94) | 3.73  (2.73) |  |  |  |
| 5-classes | 5.01  (4.20) | 16.51  (15.99) | 40.03  (41.49) | 36.04  (36.15) | 2.41  (2.16) |  |  |
| 6-classes | 0.06  (0.06) | 5.56  (4.71) | 16.39  (15.77) | 39.80  (41.46) | 35.81  (35.84) | 2.38  (2.16) |  |
| 7-classes | 0.06  (0.06) | 5.09  (4.23) | 15.51  (14.91) | 37.49  (39.49) | 29.24  (33.36) | 10.96  (6.36) | 1.66  (1.59) |

Class proportions based on the estimated posterior probabilities (most likely latent class membership in brackets). Classes ordered by mean of class.
