# Supplementary material for: Setting a standard for low reading proficiency: A comparison of the bookmark procedure and constrained mixture Rasch model
Source: PLoS One. 2021 Nov 29;16(11):e0257871. doi: 10.1371/journal.pone.0257871 (PMC8629253; doi:10.1371/journal.pone.0257871)
Supplement: S9 Table — (DOCX) [file pone.0257871.s009.docx]

**S9 Table. Item parameters among the split-half student samples.**

|  | Split-Half Student Sample 1 | | | | | | | | Split-Half Student Sample 2 | | | | | | | |
| --- | --- | --- | --- | --- | --- | --- | --- | --- | --- | --- | --- | --- | --- | --- | --- | --- |
|  | Rasch | | | | cMRM 4-class-solution | | | | Rasch | | | | cMRM 4-class-solution | | | |
| Reading item | *M* | *z* scores | SE | [95% CI] | *M* | *z* scores | SE | [95% CI] | *M* | *z*  scores | SE | [95% CI] | *M* | *z*  scores | SE | [95% CI] |
| 1_1 | -3.83 | -0.70 | 0.07 | [-3.94,-3.70] | -2.65 | -0.69 | 0.10 | [-2.84,-2.46] | -3.85 | -0.70 | 0.07 | [-3.98,-3.72] | -2.59 | -0.70 | 0.09 | [-2.77,-2.42] |
| 2_1 | -4.60 | -1.08 | 0.09 | [-4.75,-4.42] | -3.41 | -1.07 | 0.11 | [-3.63,-3.19] | -4.72 | -1.14 | 0.09 | [-4.90,-4.53] | -3.45 | -1.13 | 0.11 | [-3.67,-3.23] |
| 3_1 | -1.70 | 0.36 | 0.04 | [-1.76,-1.63] | -0.53 | 0.36 | 0.08 | [-0.69,-0.38] | -1.80 | 0.32 | 0.04 | [-1.87,-1.72] | -0.55 | 0.32 | 0.07 | [-0.69,-0.41] |
| 4_1 | -6.12 | -1.83 | 0.06 | [-6.30,-5.90] | -4.96 | -1.84 | 0.12 | [-5.20,-4.70] | -6.14 | -1.85 | 0.06 | [-6.36,-5.94] | -4.90 | -1.86 | 0.06 | [-5.14,-4.66] |
| 4_2 | -4.32 | -0.94 | 0.04 | [-4.44,-4.16] | -3.14 | -0.93 | 0.10 | [-3.36,-2.94] | -4.32 | -0.94 | 0.04 | [-4.46,-4.16] | -3.06 | -0.94 | 0.05 | [-3.26,-2.88] |
| 4_3 | -2.68 | -0.13 | 0.03 | [-2.80,-2.56] | -1.52 | -0.13 | 0.10 | [-1.70,-1.34] | -2.70 | -0.13 | 0.03 | [-2.82,-2.58] | -1.46 | -0.13 | 0.04 | [-1.64,-1.30] |
| 4_4 | 0.26 | 1.33 | 0.03 | [0.18,0.36] | 1.42 | 1.33 | 0.08 | [1.24,1.58] | 0.24 | 1.35 | 0.03 | [0.12,0.34] | 1.46 | 1.33 | 0.04 | [1.30,1.62] |
| 5_1 | -7.00 | -2.27 | 0.07 | [-7.22,-6.74] | -5.82 | -2.26 | 0.16 | [-6.12,-5.52] | -6.72 | -2.14 | 0.06 | [-6.96,-6.48] | -5.46 | -2.14 | 0.07 | [-5.74,-5.20] |
| 5_2 | -5.00 | -1.28 | 0.04 | [-5.14,-4.82] | -3.82 | -1.27 | 0.12 | [-4.04,-3.60] | -4.78 | -1.17 | 0.04 | [-4.96,-4.62] | -3.54 | -1.18 | 0.05 | [-3.74,-3.32] |
| 6_1 | -2.41 | 0.01 | 0.04 | [-2.48,-2.33] | -1.24 | 0.01 | 0.08 | [-1.40,-1.08] | -2.40 | 0.02 | 0.04 | [-2.48,-2.32] | -1.15 | 0.02 | 0.08 | [-1.30,-1.00] |
| 7_1 | -0.71 | 0.85 | 0.03 | [-0.76,-0.65] | 0.46 | 0.85 | 0.08 | [0.31,0.62] | -0.71 | 0.87 | 0.03 | [-0.77,-0.65] | 0.54 | 0.87 | 0.07 | [0.40,0.68] |
| 8_1 | -2.46 | -0.02 | 0.04 | [-2.53,-2.38] | -1.29 | -0.02 | 0.08 | [-1.45,-1.13] | -2.51 | -0.03 | 0.04 | [-2.60,-2.43] | -1.26 | -0.03 | 0.08 | [-1.41,-1.11] |
| 9_1 | -2.50 | -0.04 | 0.04 | [-2.57,-2.42] | -1.33 | -0.04 | 0.08 | [-1.49,-1.17] | -2.48 | -0.02 | 0.04 | [-2.56,-2.40] | -1.23 | -0.02 | 0.08 | [-1.38,-1.09] |
| 10_1 | 0.58 | 1.49 | 0.03 | [0.52,0.64] | 1.74 | 1.49 | 0.08 | [1.59,1.90] | 0.57 | 1.51 | 0.03 | [0.51,0.64] | 1.82 | 1.51 | 0.07 | [1.68,1.96] |
| 11_1 | -2.40 | 0.01 | 0.04 | [-2.47,-2.31] | -1.23 | 0.01 | 0.08 | [-1.39,-1.07] | -2.35 | 0.05 | 0.04 | [-2.43,-2.27] | -1.10 | 0.05 | 0.08 | [-1.25,-0.96] |
| 12_1 | -3.15 | -0.36 | 0.05 | [-3.23,-3.04] | -1.97 | -0.35 | 0.09 | [-2.14,-1.80] | -3.28 | -0.42 | 0.05 | [-3.39,-3.18] | -2.03 | -0.42 | 0.08 | [-2.18,-1.87] |
| 13_1 | -7.90 | -2.71 | 0.08 | [-8.18,-7.58] | -6.74 | -2.72 | 0.18 | [-7.08,-6.38] | -7.84 | -2.71 | 0.08 | [-8.16,-7.52] | -6.58 | -2.70 | 0.09 | [-6.92,-6.26] |
| 13_2 | -5.42 | -1.48 | 0.05 | [-5.58,-5.24] | -4.26 | -1.49 | 0.12 | [-4.48,-4.02] | -5.46 | -1.51 | 0.05 | [-5.66,-5.28] | -4.22 | -1.52 | 0.06 | [-4.44,-4.00] |
| 13_3 | -3.56 | -0.56 | 0.08 | [-3.68,-3.42] | -2.40 | -0.57 | 0.10 | [-2.58,-2.20] | -3.70 | -0.63 | 0.04 | [-3.84,-3.56] | -2.44 | -0.63 | 0.05 | [-2.64,-2.26] |
| 14_1 | -2.59 | -0.08 | 0.05 | [-2.67,-2.51] | -1.42 | -0.08 | 0.08 | [-1.58,-1.26] | -2.66 | -0.11 | 0.05 | [-2.75,-2.58] | -1.41 | -0.11 | 0.08 | [-1.56,-1.26] |
| 15_1 | -2.82 | -0.19 | 0.05 | [-2.89,-2.72] | -1.64 | -0.19 | 0.08 | [-1.81,-1.48] | -2.89 | -0.22 | 0.05 | [-2.98,-2.79] | -1.64 | -0.22 | 0.08 | [-1.79,-1.48] |
| 16_1 | -1.54 | 0.44 | 0.04 | [-1.60,-1.47] | -0.37 | 0.44 | 0.08 | [-0.53,-0.21] | -1.56 | 0.44 | 0.04 | [-1.63,-1.49] | -0.31 | 0.44 | 0.07 | [-0.46,-0.17] |
| 17_1 | -0.88 | 0.77 | 0.03 | [-0.93,-0.81] | 0.30 | 0.77 | 0.08 | [0.14,0.45] | -0.85 | 0.80 | 0.03 | [-0.92,-0.79] | 0.40 | 0.80 | 0.07 | [0.25,0.54] |

|  | Split-Half Student Sample 1 | | | | | | | | Split-Half Student Sample 2 | | | | | | | |
| --- | --- | --- | --- | --- | --- | --- | --- | --- | --- | --- | --- | --- | --- | --- | --- | --- |
|  | Rasch | | | | cMRM 4-class-solution | | | | Rasch | | | | cMRM 4-class-solution | | | |
| Reading item | *M* | *z*  scores | SE | [95% CI] | *M* | *z*  scores | SE | [95% CI] | *M* | *z*  scores | SE | [95% CI] | *M* | *z*  scores | SE | [95% CI] |
| 18_1 | -2.39 | 0.02 | 0.04 | [-2.46,-2.30] | -1.22 | 0.02 | 0.08 | [-1.38,-1.05] | -2.40 | 0.02 | 0.04 | [-2.48,-2.31] | -1.15 | 0.02 | 0.08 | [-1.3,-1.00] |
| 19_1 | -1.42 | 0.50 | 0.04 | [-1.48,-1.35] | -0.25 | 0.50 | 0.08 | [-0.40,-0.09] | -1.46 | 0.49 | 0.04 | [-1.53,-1.39] | -0.21 | 0.49 | 0.07 | [-0.35,-0.07] |
| 20_1 | -1.01 | 0.70 | 0.03 | [-1.07,-0.94] | 0.16 | 0.70 | 0.08 | [0.01,0.32] | -0.99 | 0.73 | 0.03 | [-1.05,-0.92] | 0.26 | 0.73 | 0.07 | [0.12,0.40] |
| 21_1 | -1.72 | 0.35 | 0.04 | [-1.78,-1.64] | -0.55 | 0.35 | 0.08 | [-0.70,-0.39] | -1.80 | 0.32 | 0.04 | [-1.87,-1.72] | -0.55 | 0.32 | 0.07 | [-0.69,-0.41] |
| 22_1 | -2.13 | 0.15 | 0.04 | [-2.2,-2.05] | -0.96 | 0.15 | 0.08 | [-1.12,-0.80] | -2.13 | 0.16 | 0.04 | [-2.21,-2.05] | -0.88 | 0.16 | 0.07 | [-1.03,-0.74] |
| 23_1 | -0.76 | 0.83 | 0.03 | [-0.81,-0.69] | 0.41 | 0.83 | 0.08 | [0.26,0.57] | -0.80 | 0.82 | 0.03 | [-0.87,-0.73] | 0.45 | 0.83 | 0.07 | [0.31,0.59] |
| 24_1 | -3.66 | -0.61 | 0.04 | [-3.78,-3.52] | -2.50 | -0.62 | 0.05 | [-2.68,-2.30] | -3.76 | -0.66 | 0.08 | [-3.90,-3.60] | -2.50 | -0.66 | 0.05 | [-2.70,-2.32] |
| 24_2 | -2.42 | 0.00 | 0.03 | [-2.54,-2.30] | -1.26 | 0.00 | 0.05 | [-1.44,-1.08] | -2.54 | -0.05 | 0.06 | [-2.66,-2.40] | -1.28 | -0.04 | 0.04 | [-1.46,-1.12] |
| 25_1 | -0.16 | 1.12 | 0.03 | [-0.22,-0.10] | 1.01 | 1.12 | 0.08 | [0.86,1.16] | -0.19 | 1.13 | 0.03 | [-0.26,-0.13] | 1.06 | 1.13 | 0.07 | [0.92,1.20] |
| 26_1 | -0.66 | 0.88 | 0.03 | [-0.72,-0.60] | 0.51 | 0.88 | 0.08 | [0.35,0.66] | -0.68 | 0.88 | 0.04 | [-0.75,-0.61] | 0.57 | 0.89 | 0.07 | [0.43,0.71] |
| 27_1 | -0.54 | 0.94 | 0.04 | [-0.60,-0.47] | 0.63 | 0.94 | 0.08 | [0.47,0.78] | -0.47 | 0.99 | 0.04 | [-0.54,-0.40] | 0.78 | 0.99 | 0.07 | [0.64,0.92] |
| 28_1 | 0.14 | 1.27 | 0.04 | [0.08,0.21] | 1.30 | 1.27 | 0.08 | [1.15,1.46] | 0.12 | 1.29 | 0.04 | [0.05,0.18] | 1.36 | 1.28 | 0.07 | [1.22,1.50] |
| 29_1 | -0.02 | 1.19 | 0.04 | [-0.07,0.05] | 1.15 | 1.19 | 0.08 | [1.00,1.31] | -0.10 | 1.18 | 0.04 | [-0.17,-0.03] | 1.15 | 1.18 | 0.07 | [1.01,1.29] |
| 30_1 | -0.77 | 0.82 | 0.04 | [-0.83,-0.70] | 0.40 | 0.82 | 0.08 | [0.24,0.55] | -0.76 | 0.84 | 0.04 | [-0.83,-0.69] | 0.49 | 0.85 | 0.07 | [0.35,0.63] |
| 31_1 | -1.95 | 0.24 | 0.04 | [-2.02,-1.87] | -0.78 | 0.24 | 0.08 | [-0.94,-0.62] | -2.03 | 0.21 | 0.04 | [-2.12,-1.94] | -0.78 | 0.21 | 0.08 | [-0.93,-0.63] |

**S9 Table (continued)**

*M* = Mean, SE = Standard error, 95% CI = 95% confidence interval. Difficulty parameters were transformed into standardized scores (z-scores) with a mean of 0 and a standard deviation of 1. The difficulty parameters of the cMRMs were constrained to be equal across the classes. Pearson’s correlation coefficients between the respective Rasch model and cMRM were *r* =1.00 for both split-half student samples. Polytomous reading items with more than two categories are marked with the number 2 or higher after the underscore of the reading item (e.g., 4_2, 11_4). To assess the robustness of the item parameter estimates, the NEPS scaling model was rewritten for partial-credit items with βij = 2δij. This changes nothing relating to the fit of the model and the WLE estimates of θ are the same, but it posits person and item parameters on the same scale.
