# Supplementary material for: Setting a standard for low reading proficiency: A comparison of the bookmark procedure and constrained mixture Rasch model
Source: PLoS One. 2021 Nov 29;16(11):e0257871. doi: 10.1371/journal.pone.0257871 (PMC8629253; doi:10.1371/journal.pone.0257871)
Supplement: S10 Table — (DOCX) [file pone.0257871.s010.docx]

**S10 Table. Item parameters among the adult samples.**

|  | Adult Sample 1 | | | | | | | | Adult Sample 2 | | | | | | | |
| --- | --- | --- | --- | --- | --- | --- | --- | --- | --- | --- | --- | --- | --- | --- | --- | --- |
|  | Rasch | | | | cMRM 3-class-solution | | | | Rasch | | | | cMRM 3-class-solution | | | |
| Reading item | *M* | *z*  scores | SE | [95% CI] | *M* | *z*  scores | SE | [95% CI] | *M* | *z*  scores | SE | [95% CI] | *M* | *z*  scores | SE | [95% CI] |
| 1_1 | -3.65 | -1.03 | 0.07 | [-3.79,-3.51] | -1.62 | -1.02 | 0.09 | [-1.79,-1.44] | -3.37 | -1.17 | 0.09 | [-3.53,-3.20] | -4.62 | -1.16 | 0.10 | [-4.81,-4.43] |
| 2_1 | -6.24 | -2.62 | 0.07 | [-6.52,-5.96] | -4.24 | -2.64 | 0.08 | [-4.54,-3.94] | -5.18 | -2.35 | 0.08 | [-5.46,-4.88] | -6.46 | -2.36 | 0.08 | [-6.76,-6.16] |
| 2_2 | -2.76 | -0.49 | 0.04 | [-2.92,-2.60] | -0.78 | -0.50 | 0.05 | [-0.96,-0.58] | -2.20 | -0.41 | 0.05 | [-2.38,-2.00] | -3.48 | -0.41 | 0.05 | [-3.70,-3.28] |
| 3_1 | -3.36 | -0.86 | 0.07 | [-3.49,-3.23] | -1.33 | -0.84 | 0.08 | [-1.49,-1.17] | -2.81 | -0.81 | 0.07 | [-2.96,-2.67] | -4.07 | -0.80 | 0.09 | [-4.24,-3.90] |
| 4_1 | -2.05 | -0.05 | 0.05 | [-2.14,-1.96] | -0.05 | -0.05 | 0.07 | [-0.19,0.09] | -1.41 | 0.10 | 0.05 | [-1.52,-1.31] | -2.69 | 0.11 | 0.08 | [-2.84,-2.54] |
| 5_1 | -4.42 | -1.50 | 0.05 | [-4.60,-4.22] | -2.42 | -1.51 | 0.06 | [-2.64,-2.20] | -3.76 | -1.43 | 0.06 | [-3.98,-3.54] | -5.04 | -1.43 | 0.06 | [-5.28,-4.82] |
| 5_2 | -0.82 | 0.70 | 0.03 | [-0.94,-0.70] | 1.16 | 0.70 | 0.04 | [1.00,1.32] | -0.36 | 0.78 | 0.04 | [-0.54,-0.20] | -1.68 | 0.77 | 0.05 | [-1.86,-1.48] |
| 6_1 | -3.58 | -0.99 | 0.07 | [-3.71,-3.44] | -1.54 | -0.97 | 0.09 | [-1.71,-1.37] | -3.25 | -1.09 | 0.08 | [-3.41,-3.09] | -4.51 | -1.08 | 0.10 | [-4.70,-4.32] |
| 7_1 | -2.60 | -0.39 | 0.05 | [-2.70,-2.50] | -0.59 | -0.38 | 0.08 | [-0.74,-0.44] | -2.21 | -0.42 | 0.06 | [-2.33,-2.09] | -3.49 | -0.42 | 0.08 | [-3.64,-3.33] |
| 8_1 | -3.04 | -0.66 | 0.06 | [-3.16,-2.93] | -1.02 | -0.65 | 0.08 | [-1.18,-0.86] | -2.61 | -0.68 | 0.07 | [-2.74,-2.48] | -3.88 | -0.67 | 0.08 | [-4.04,-3.71] |
| 9_1 | -2.50 | -0.33 | 0.05 | [-2.60,-2.40] | -0.49 | -0.32 | 0.08 | [-0.64,-0.34] | -2.20 | -0.41 | 0.06 | [-2.32,-2.08] | -3.47 | -0.40 | 0.08 | [-3.63,-3.32] |
| 10_1 | -2.20 | -0.15 | 0.05 | [-2.30,-2.11] | -0.20 | -0.14 | 0.07 | [-0.35,-0.06] | -2.02 | -0.29 | 0.06 | [-2.13,-1.90] | -3.29 | -0.28 | 0.08 | [-3.45,-3.14] |
| 11_1 | -4.60 | -1.61 | 0.05 | [-4.80,-4.40] | -2.60 | -1.62 | 0.06 | [-2.84,-2.38] | -3.90 | -1.52 | 0.06 | [-4.12,-3.66] | -5.18 | -1.52 | 0.06 | [-5.44,-4.94] |
| 11_2 | -3.16 | -0.73 | 0.04 | [-3.32,-3.00] | -1.18 | -0.75 | 0.05 | [-1.38,-0.98] | -2.68 | -0.72 | 0.05 | [-2.88,-2.48] | -3.98 | -0.74 | 0.05 | [-4.20,-3.78] |
| 11_3 | -2.06 | -0.06 | 0.04 | [-2.22,-1.92] | -0.08 | -0.07 | 0.05 | [-0.26,0.10] | -1.64 | -0.05 | 0.05 | [-1.82,-1.46] | -2.94 | -0.05 | 0.05 | [-3.14,-2.76] |
| 11_4 | -0.62 | 0.82 | 0.03 | [-0.76,-0.50] | 1.34 | 0.81 | 0.04 | [1.18,1.50] | -0.34 | 0.80 | 0.04 | [-0.52,-0.16] | -1.64 | 0.80 | 0.05 | [-1.84,-1.46] |
| 11_5 | 1.14 | 1.90 | 0.03 | [1.02,1.28] | 3.10 | 1.90 | 0.04 | [2.94,3.26] | 1.32 | 1.88 | 0.05 | [1.14,1.48] | -0.02 | 1.86 | 0.05 | [-0.20,0.18] |
| 12_1 | -1.16 | 0.49 | 0.04 | [-1.24,-1.09] | 0.83 | 0.49 | 0.07 | [0.69,0.97] | -0.94 | 0.41 | 0.05 | [-1.05,-0.84] | -2.23 | 0.41 | 0.08 | [-2.38,-2.08] |
| 13_1 | -2.04 | -0.05 | 0.05 | [-2.13,-1.95] | -0.04 | -0.04 | 0.07 | [-0.18,0.10] | -1.70 | -0.09 | 0.06 | [-1.81,-1.59] | -2.98 | -0.08 | 0.08 | [-3.13,-2.83] |
| 14_1 | -1.71 | 0.15 | 0.04 | [-1.80,-1.63] | 0.28 | 0.16 | 0.07 | [0.14,0.42] | -1.52 | 0.03 | 0.06 | [-1.63,-1.41] | -2.80 | 0.04 | 0.08 | [-2.95,-2.65] |
| 15_1 | -0.45 | 0.93 | 0.04 | [-0.52,-0.37] | 1.54 | 0.93 | 0.07 | [1.41,1.68] | -0.11 | 0.95 | 0.05 | [-0.20,-0.01] | -1.39 | 0.96 | 0.07 | [-1.53,-1.26] |
| 16_1 | -2.23 | -0.16 | 0.05 | [-2.33,-2.14] | -0.23 | -0.16 | 0.07 | [-0.38,-0.09] | -1.94 | -0.24 | 0.06 | [-2.06,-1.82] | -3.22 | -0.24 | 0.08 | [-3.38,-3.07] |
| 17_1 | -1.99 | -0.02 | 0.05 | [-2.08,-1.90] | 0.01 | -0.01 | 0.07 | [-0.13,0.15] | -1.59 | -0.02 | 0.06 | [-1.71,-1.48] | -2.88 | -0.02 | 0.08 | [-3.03,-2.73] |

**S10 Table (continued).**

|  | Adult Sample 1 | | | | | | | | Adult Sample 2 | | | | | | | |
| --- | --- | --- | --- | --- | --- | --- | --- | --- | --- | --- | --- | --- | --- | --- | --- | --- |
|  | Rasch | | | | cMRM 3-class-solution | | | | Rasch | | | | cMRM 3-class-solution | | | |
| Reading item | *M* | *z*  scores | SE | [95% CI] | *M* | *z*  scores | SE | [95% CI] | *M* | *z*  scores | SE | [95% CI] | *M* | *z*  scores | SE | [95% CI] |
| 18_1 | -1.14 | 0.50 | 0.04 | [-1.22,-1.06] | 0.85 | 0.51 | 0.07 | [0.71,0.99] | -0.70 | 0.56 | 0.05 | [-0.80,-0.60] | -1.99 | 0.57 | 0.08 | [-2.13,-1.84] |
| 19_1 | -3.48 | -0.93 | 0.04 | [-3.64,-3.30] | -1.48 | -0.93 | 0.05 | [-1.70,-1.28] | -2.98 | -0.92 | 0.06 | [-3.2,-2.78] | -4.28 | -0.93 | 0.06 | [-4.52,-4.06] |
| 19_2 | 0.70 | 1.63 | 0.04 | [0.58,0.84] | 2.66 | 1.62 | 0.04 | [2.50,2.84] | 1.32 | 1.88 | 0.05 | [1.14,1.50] | 0.00 | 1.87 | 0.05 | [-0.20,0.20] |
| 20_1 | -0.25 | 1.05 | 0.04 | [-0.33,-0.17] | 1.74 | 1.06 | 0.07 | [1.60,1.88] | 0.01 | 1.02 | 0.05 | [-0.09,0.11] | -1.28 | 1.03 | 0.08 | [-1.43,-1.13] |
| 21_1 | -3.02 | -0.65 | 0.04 | [-3.18,-2.86] | -1.04 | -0.66 | 0.05 | [-1.24,-0.86] | -2.40 | -0.54 | 0.05 | [-2.60,-2.20] | -3.70 | -0.55 | 0.05 | [-3.92,-3.50] |
| 21_2 | -0.30 | 1.02 | 0.03 | [-0.44,-0.18] | 1.66 | 1.01 | 0.04 | [1.5,1.82] | 0.12 | 1.10 | 0.04 | [-0.06,0.30] | -1.20 | 1.09 | 0.05 | [-1.38,-1.00] |
| 22_1 | -1.73 | 0.14 | 0.05 | [-1.82,-1.64] | 0.26 | 0.14 | 0.08 | [0.12,0.41] | -1.34 | 0.15 | 0.06 | [-1.45,-1.22] | -2.62 | 0.16 | 0.08 | [-2.78,-2.47] |
| 23_1 | -2.85 | -0.54 | 0.06 | [-2.97,-2.73] | -0.84 | -0.54 | 0.08 | [-1.01,-0.68] | -2.17 | -0.39 | 0.07 | [-2.29,-2.04] | -3.44 | -0.38 | 0.08 | [-3.60,-3.29] |
| 24_1 | -2.11 | -0.09 | 0.05 | [-2.21,-2.01] | -0.12 | -0.09 | 0.08 | [-0.27,0.04] | -1.82 | -0.16 | 0.06 | [-1.94,-1.69] | -3.10 | -0.16 | 0.08 | [-3.26,-2.94] |
| 25_1 | 0.51 | 1.51 | 0.04 | [0.43,0.59] | 2.49 | 1.52 | 0.07 | [2.35,2.63] | 0.73 | 1.49 | 0.06 | [0.62,0.84] | -0.57 | 1.50 | 0.07 | [-0.71,-0.43] |
| 26_1 | -2.79 | -0.51 | 0.07 | [-2.94,-2.65] | -0.79 | -0.51 | 0.09 | [-0.96,-0.62] | -2.71 | -0.74 | 0.09 | [-2.89,-2.53] | -3.97 | -0.73 | 0.10 | [-4.18,-3.77] |
| 27_1 | -2.22 | -0.16 | 0.05 | [-2.40,-2.04] | -0.24 | -0.17 | 0.06 | [-0.46,-0.02] | -1.84 | -0.18 | 0.06 | [-2.08,-1.62] | -3.16 | -0.20 | 0.06 | [-3.40,-2.90] |
| 28_1 | -1.55 | 0.25 | 0.06 | [-1.66,-1.44] | 0.44 | 0.25 | 0.08 | [0.29,0.60] | -1.39 | 0.11 | 0.07 | [-1.53,-1.25] | -2.67 | 0.12 | 0.09 | [-2.85,-2.50] |
| 29_1 | -2.08 | -0.07 | 0.05 | [-2.28,-1.88] | -0.12 | -0.09 | 0.06 | [-0.34,0.12] | -1.90 | -0.22 | 0.07 | [-2.18,-1.64] | -3.22 | -0.24 | 0.07 | [-3.50,-2.94] |
| 29_2 | 1.74 | 2.27 | 0.05 | [1.56,1.94] | 3.68 | 2.25 | 0.05 | [3.46,3.88] | 2.10 | 2.38 | 0.07 | [1.86,2.36] | 0.76 | 2.37 | 0.07 | [0.48,1.02] |
| 30_1 | 0.14 | 1.29 | 0.06 | [0.03,0.26] | 2.12 | 1.29 | 0.08 | [1.96,2.28] | 0.29 | 1.21 | 0.08 | [0.13,0.44] | -1.02 | 1.20 | 0.09 | [-1.19,-0.84] |

*M* = Mean, SE = Standard error, 95% CI = 95% confidence interval. The difficulty parameters of the cMRMs were constrained to be equal across the classes. Difficulty parameters were transformed into standardized scores (z-scores) with a mean of 0 and a standard deviation of 1. Pearson’s correlation coefficients between the respective Rasch model and cMRM were *r* =1.00 for both adult samples. Polytomous reading items with more than two categories are marked with the number 2 or higher after the underscore of the reading item (e.g. 5_2, 11_4). To assess the robustness of the item parameter estimates, the NEPS scaling model was rewritten for partial-credit items with βij = 2δij. This changes nothing relating to the fit of the model and the WLE estimates of θ are the same, but it posits person and item parameters on the same scale.
