# Supplementary material for: Setting a standard for low reading proficiency: A comparison of the bookmark procedure and constrained mixture Rasch model
Source: PLoS One. 2021 Nov 29;16(11):e0257871. doi: 10.1371/journal.pone.0257871 (PMC8629253; doi:10.1371/journal.pone.0257871)
Supplement: S11 Table — (DOCX) [file pone.0257871.s011.docx]

**S11 Table. Descriptive statistics by latent classes on reading competence among the split-half student samples.**

|  |  | Split-Half Student Sample 1 | | | | | |  | Split-Half Student Sample 2 | | | | | |
| --- | --- | --- | --- | --- | --- | --- | --- | --- | --- | --- | --- | --- | --- | --- |
|  | *N* | *M_WLE_* | *M_WLE_*  *z-score* | *M_F_*  *z-score* | *SD* | *Min* | *Max* | *N* | *M* | *M_WLE_*  *z-score* | *M_F_*  *z-score* | *SD* | *Min* | *Max* |
| Class 1 | 486 | -2.38 | -1.12 | -1.14 | 0.41 | -4.30 | -1.73 | 389 | -2.49 | -1.12 | -1.15 | 0.42 | -4.75 | -1.88 |
| Class 2 | 1,508 | -1.24 | -0.40 | -0.39 | 0.34 | -2.30 | -0.67 | 1,462 | -1.29 | 0.39 | 0.38 | 0.34 | -2.22 | -0.73 |
| Class 3 | 2,678 | -0.11 | 0.30 | 0.33 | 0.36 | -1.11 | 0.60 | 2,781 | -0.15 | 0.30 | 0.32 | 0.38 | -1.20 | 0.56 |
| Class 4 | 2,277 | 1.35 | 1.22 | 1.20 | 0.72 | -0.26 | 3.30 | 2,316 | 1.37 | 1.22 | 1.20 | 0.74 | -0.46 | 3.30 |

M_WLE =_ mean weighted maximum likelihood estimates (WLE); M_F_ = mean factor score estimates. Mean person abilities were transformed into standardized scores (z-scores) with a mean of 0 and a standard deviation of 1.
