# Supplementary material for: Setting a standard for low reading proficiency: A comparison of the bookmark procedure and constrained mixture Rasch model
Source: PLoS One. 2021 Nov 29;16(11):e0257871. doi: 10.1371/journal.pone.0257871 (PMC8629253; doi:10.1371/journal.pone.0257871)
Supplement: S12 Table — (DOCX) [file pone.0257871.s012.docx]

**S12 Table. Descriptive statistics by latent classes on reading competence among the adult samples.**

|  |  | Adult Sample 1 | | | | | |  | Adult Sample 2 | | | | | |
| --- | --- | --- | --- | --- | --- | --- | --- | --- | --- | --- | --- | --- | --- | --- |
|  | *N* | *M_WLE_* | *M_WLE_*  *z-score* | *M_F_*  *z-score* | *SD* | *Min* | *Max* | *N* | *M* | *M_WLE_*  *z-score* | *M_F_*  *z-score* | *SD* | *Min* | *Max* |
| Class 1 | 771 | -2.03 | -0.98 | -0.99 | 0.61 | -5.70 | -1.09 | 492 | -2.40 | -0.98 | -0.99 | 0.58 | -5.24 | -1.43 |
| Class 2 | 2,579 | -0.47 | -0.04 | -0.02 | 0.50 | -2.88 | 0.53 | 1,458 | -0.78 | -0.03 | -0.03 | 0.50 | -2.39 | 0.18 |
| Class 3 | 1,985 | 1.29 | 1.02 | 1.01 | 0.93 | -0.76 | 4.49 | 1,195 | 0.99 | 1.01 | 1.01 | 0.90 | -0.58 | 4.43 |

M_WLE =_ mean weighted maximum likelihood estimates (WLE); M_F_ = mean factor score estimates. Mean person abilities were transformed into standardized scores (z-scores) with a mean of 0 and a standard deviation of 1.
