# Supplementary figures and images for: Setting a standard for low reading proficiency: A comparison of the bookmark procedure and constrained mixture Rasch model
Source: PLoS One. 2021 Nov 29;16(11):e0257871. doi: 10.1371/journal.pone.0257871 (PMC8629253; doi:10.1371/journal.pone.0257871)

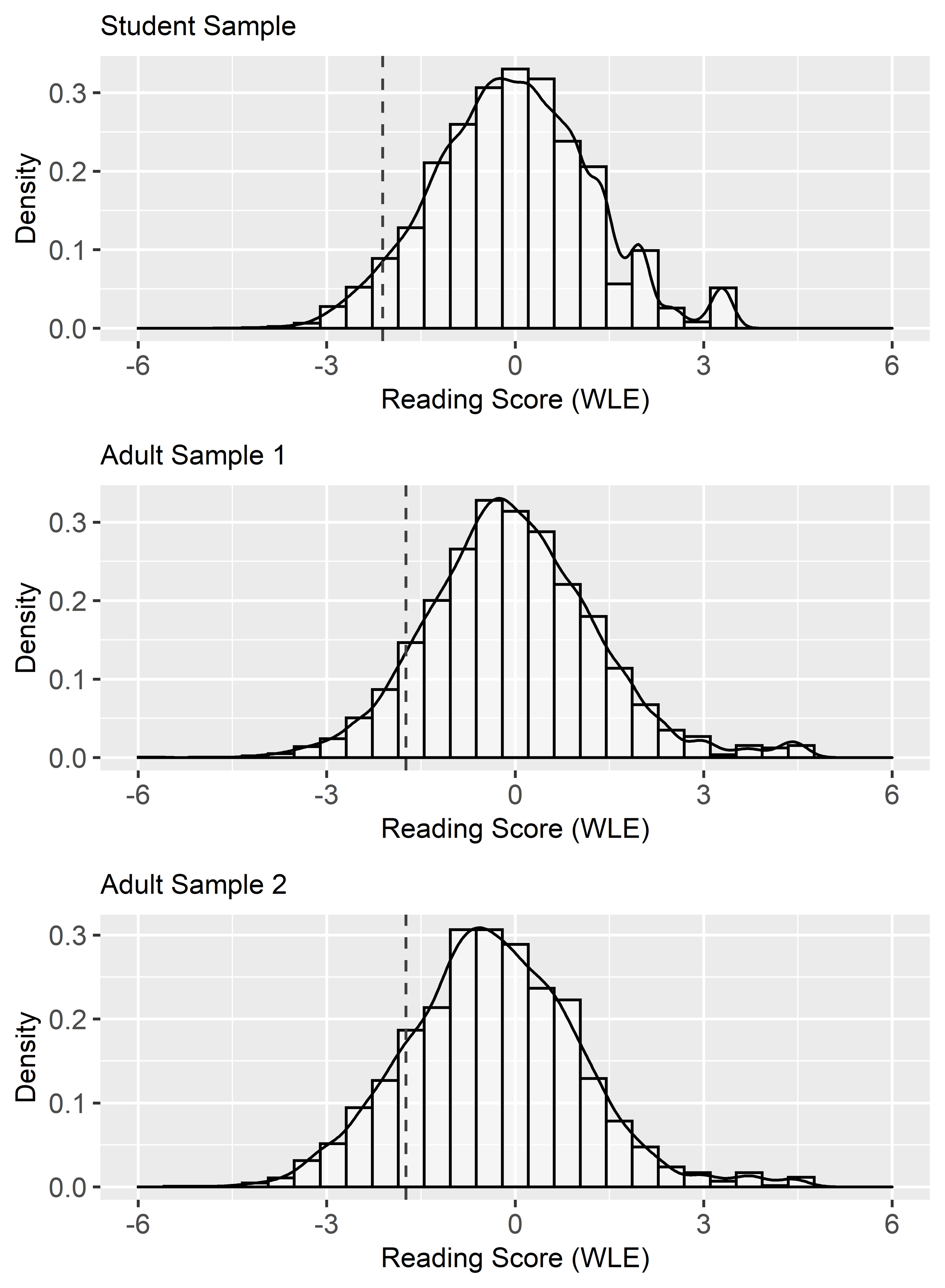

Supplement: S1 Fig — The density plots display the reading ability distribution for the different samples according to the respective Rasch models. The dashed vertical lines mark the cut scores for the Bookmark procedure. (TIF) [file pone.0257871.s013.tif]

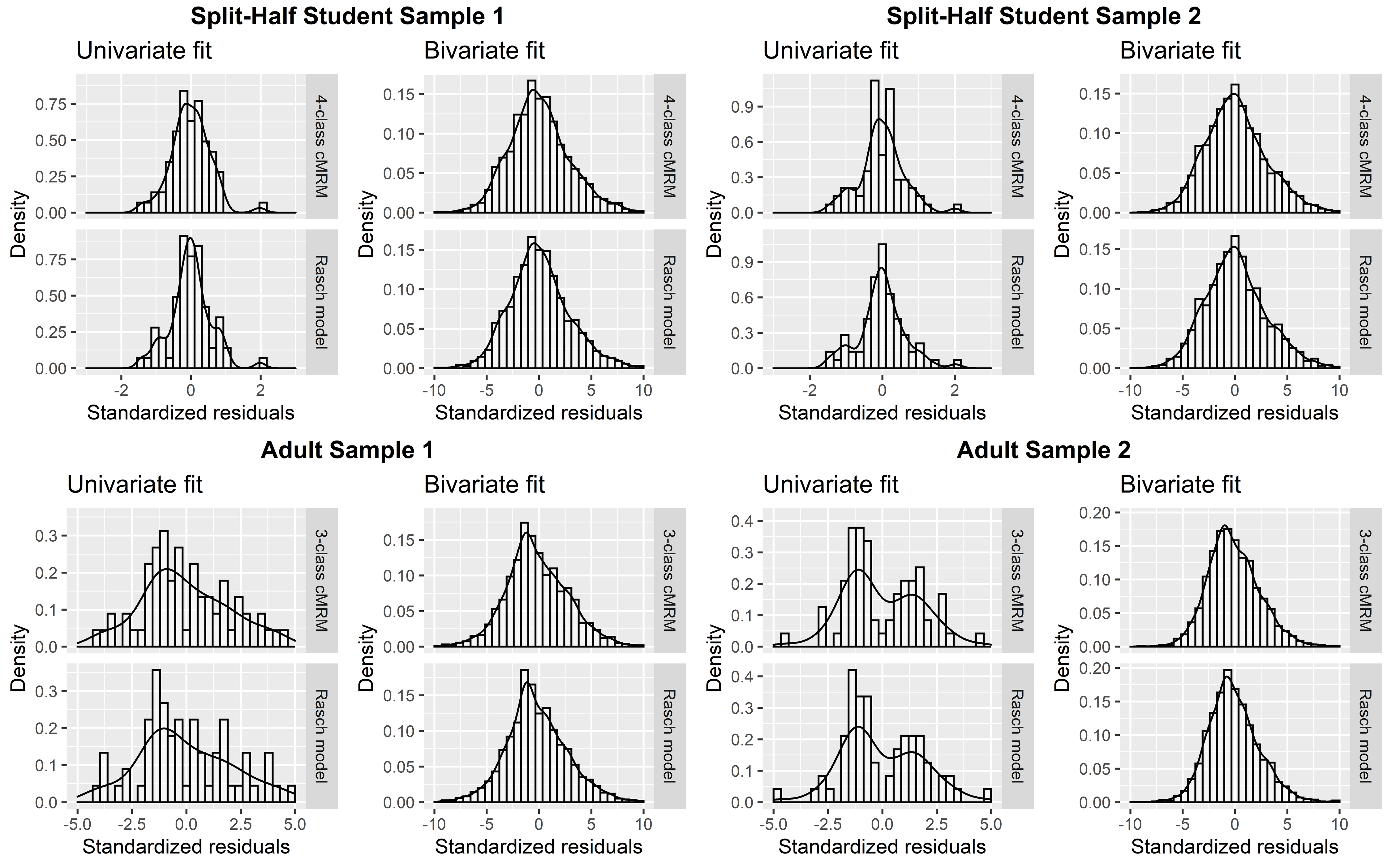

Supplement: S2 Fig — The figure shows the univariate and bivariate model fit information for the respective Rasch models and final cMRM class solutions among the two split-half student samples and two adult samples. The univariate model fit compared predicted and observed frequencies of responses for all reading items marginally. The bivariate model fits compared predicted and observed frequencies of responses for each pair of reading items. Given the large sample size, standardized residuals > |6| were considered as having a noticeable item misfit. Both model approaches, the Rasch model and cMRM showed a comparable model fit and therefore indicate that both approaches represent an acceptable measurement model. For univariate model fit, 0.00% to 2.90% items showed a noticeable misfit. For bivariate model fit, 1.79% to 5.02% items showed a noticeable misfit. Note: Some local dependence is induced by the testlet design in NEPS, that is, that the reading tests consisted of five texts, each with a set of reading items referring to the same stimulus, and by specific response formats that consist of item bundles referring to a common stimulus. (TIF) [file pone.0257871.s014.tif]

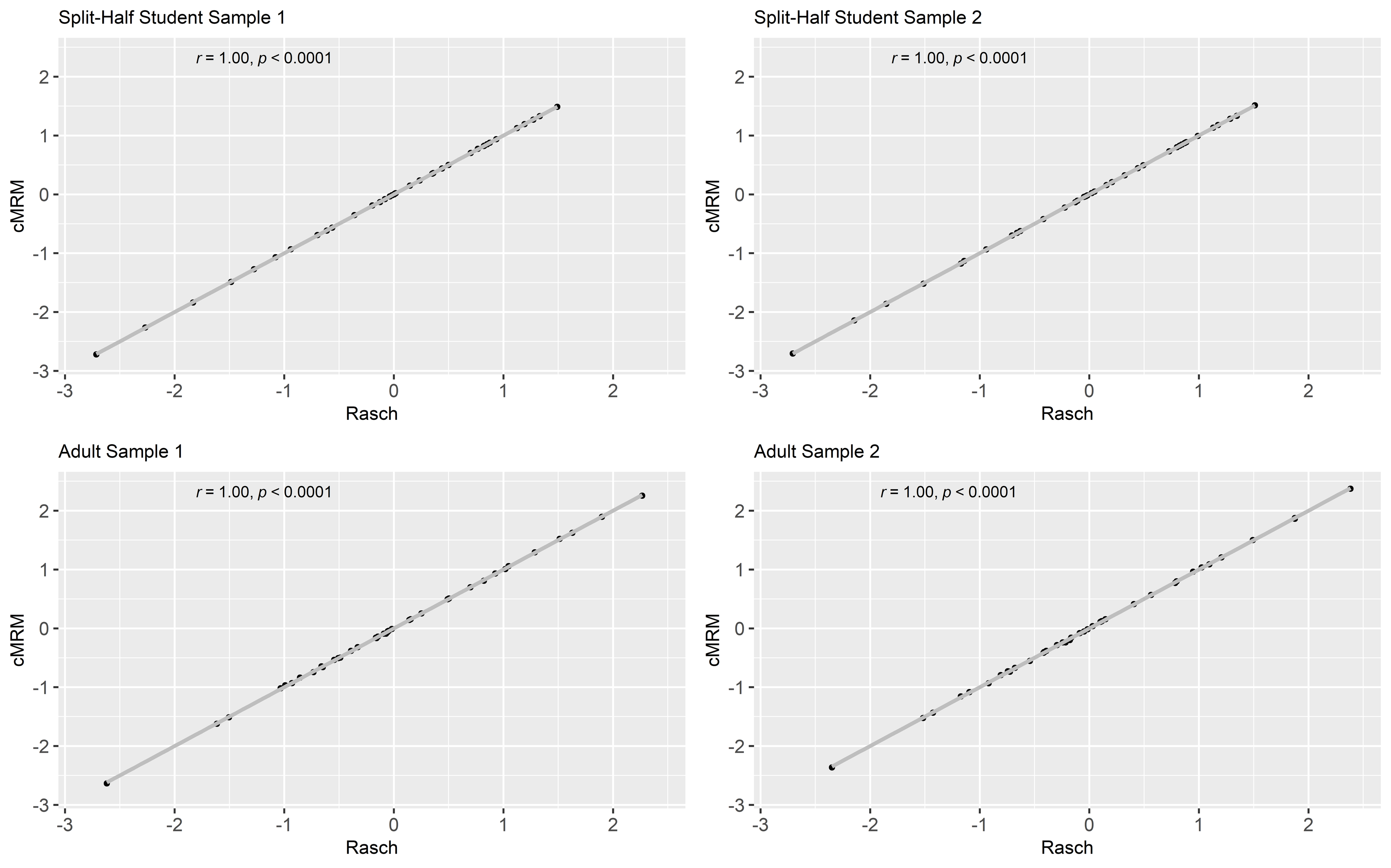

Supplement: S3 Fig — (TIF) [file pone.0257871.s015.tif]

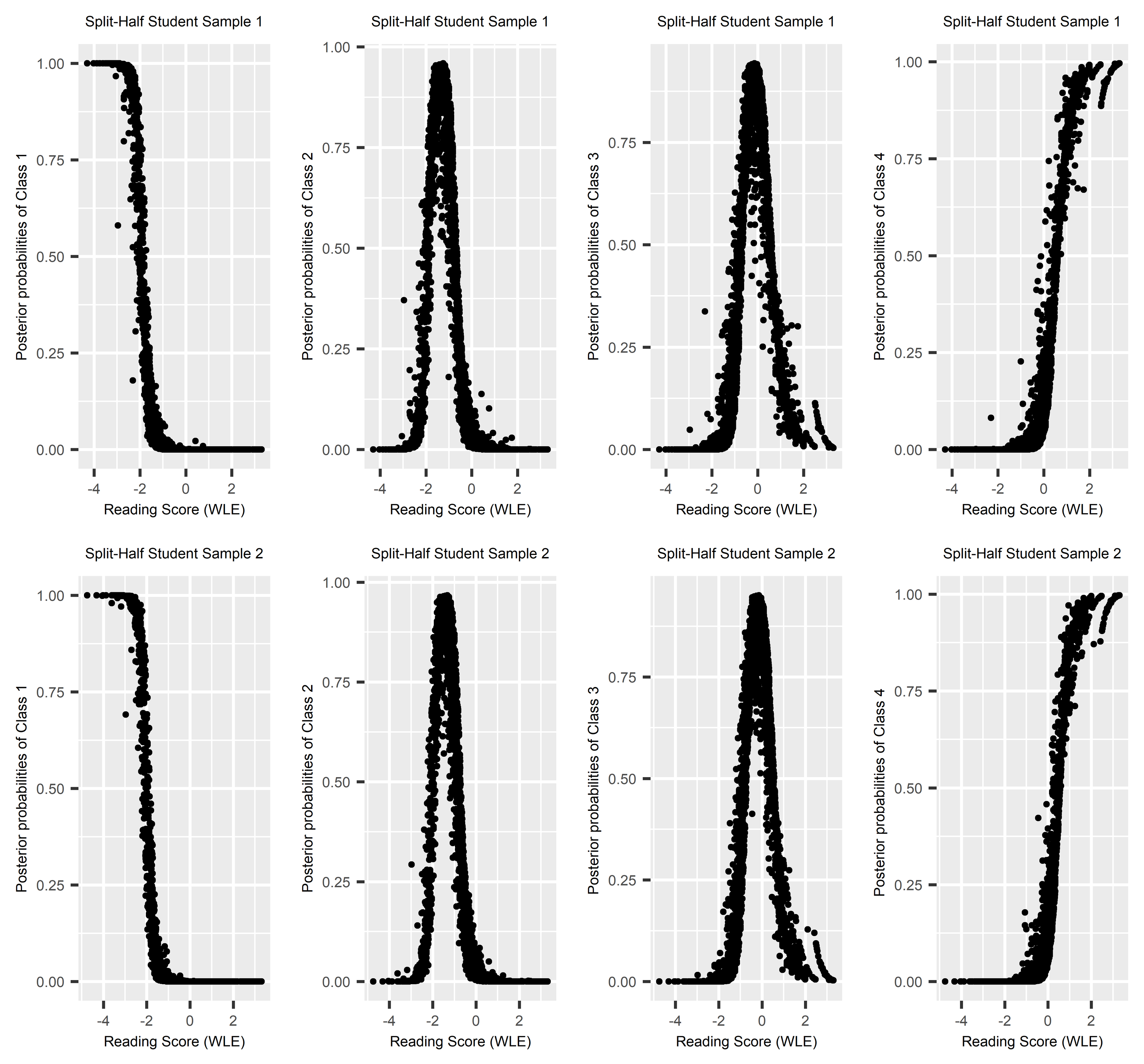

Supplement: S4 Fig — The figure shows the distribution of the most likely class membership depending on the WLE estimates of the person parameters by the two split-half student sample. As can be seen, there is a clear association between class membership and the WLE estimates; less competent persons are primarily belong to class 1 (here: low literacy group). (TIF) [file pone.0257871.s016.tif]

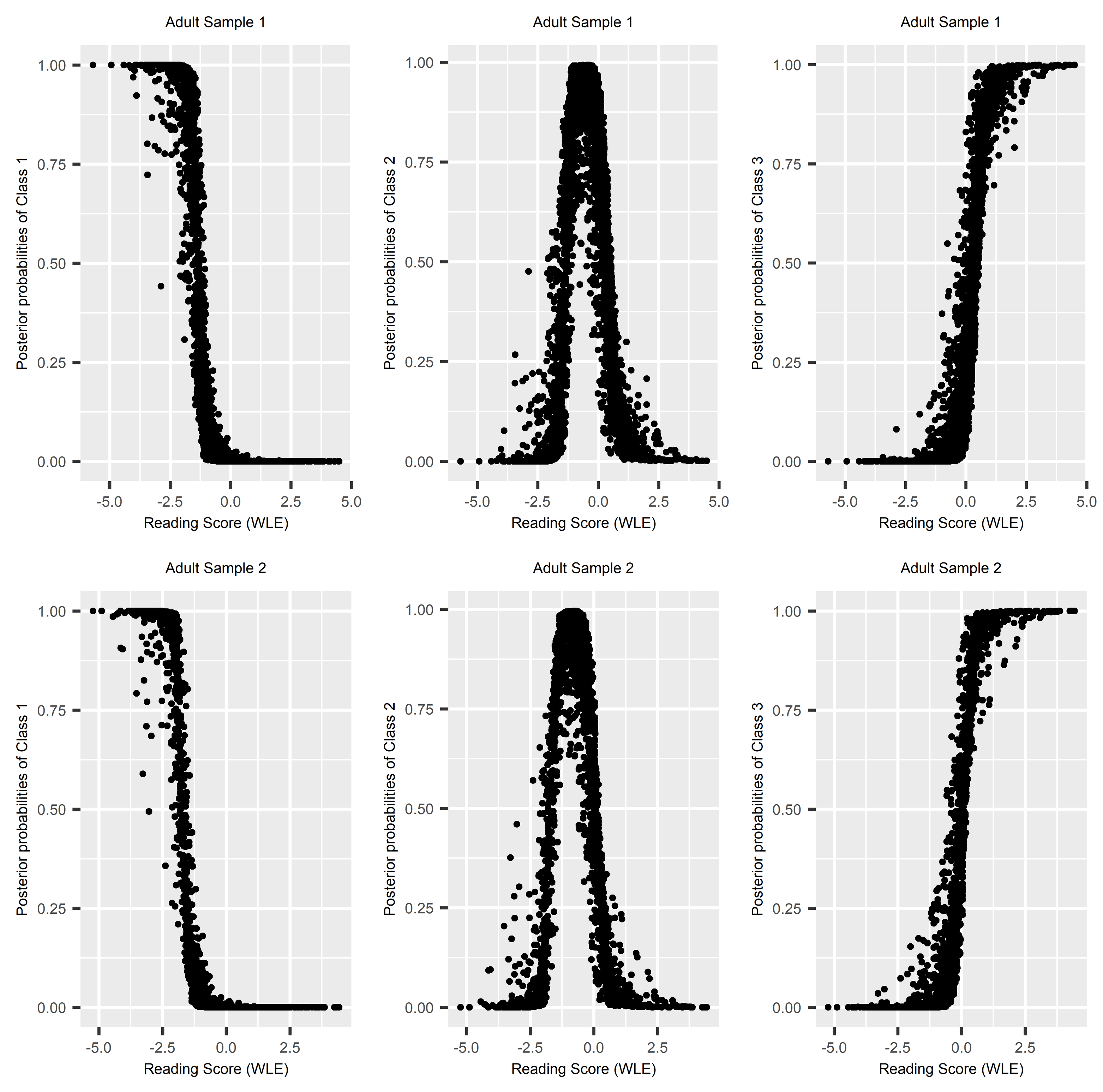

Supplement: S5 Fig — The figure shows the distribution of the most likely class membership depending on the WLE estimates of the person parameters by the two adult sample. As can be seen, there is a clear association between class membership and the WLE estimates; less competent persons are primarily belong to class 1 (here: low literacy group). (TIF) [file pone.0257871.s017.tif]
